# The F plasmid conjutome: the repertoire of *E. coli* proteins translocated through an F-encoded type IV secretion system

**DOI:** 10.1128/msphere.00354-24

**Published:** 2024-06-28

**Authors:** Abu Amar M. Al Mamun, Kimberley Kissoon, Yang Grace Li, Erin Hancock, Peter J. Christie

**Affiliations:** 1 Department of Microbiology and Molecular Genetics, McGovern Medical School at UTHealth, Houston, Texas, USA; University of Kentucky College of Medicine, Lexington, Kentucky, USA

**Keywords:** conjugation, horizontal DNA transfer, type IV secretion, protein translocation, F plasmid, effector translocator

## Abstract

Bacterial conjugation systems pose a major threat to human health through their widespread dissemination of mobile genetic elements (MGEs) carrying cargoes of antibiotic resistance genes. Using the Cre Recombinase Assay for Translocation (CRAfT), we recently reported that the IncFV pED208 conjugation system also translocates at least 16 plasmid-encoded proteins to recipient bacteria. Here, we deployed a high-throughput CRAfT screen to identify the repertoire of chromosomally encoded protein substrates of the pED208 system. We identified 32 substrates encoded by the *Escherichia coli* W3110 genome with functions associated with (i) DNA/nucleotide metabolism, (ii) stress tolerance/physiology, (iii) transcriptional regulation, or (iv) toxin inhibition. The respective gene deletions did not impact pED208 transfer proficiencies, nor did Group 1 (DNA/nucleotide metabolism) mutations detectably alter the SOS response elicited in new transconjugants upon acquisition of pED208. However, MC4100(pED208) donor cells intrinsically exhibit significantly higher SOS activation than plasmid-free MC4100 cells, and this plasmid carriage-induced stress response is further elevated in donor cells deleted of several Group 1 genes. Among 10 characterized substrates, we gained evidence of C-terminal or internal translocation signals that could function independently or synergistically for optimal protein transfer. Remarkably, nearly all tested proteins were also translocated through the IncN pKM101 and IncP RP4 conjugation systems. This repertoire of *E. coli* protein substrates, here termed the F plasmid “conjutome,” is thus characterized by functions of potential benefit to new transconjugants, diverse TSs, and the capacity for promiscuous transfer through heterologous conjugation systems.

**IMPORTANCE:**

Conjugation systems comprise a major subfamily of the type IV secretion systems (T4SSs) and are the progenitors of a second large T4SS subfamily dedicated to translocation of protein effectors. This study examined the capacity of conjugation machines to function as protein translocators. Using a high-throughput reporter screen, we determined that 32 chromosomally encoded proteins are delivered through an F plasmid conjugation system. The translocated proteins potentially enhance the establishment of the co-transferred F plasmid or mitigate mating-induced stresses. Translocation signals located C-terminally or internally conferred substrate recognition by the F system and, remarkably, many substrates also were translocated through heterologous conjugation systems. Our findings highlight the plasticity of conjugation systems in their capacities to co-translocate DNA and many protein substrates.

## INTRODUCTION

The bacterial type IV secretion systems (T4SSs) comprise a superfamily of translocators functioning mainly as conjugation machines or as effector translocators (1). Conjugation systems mediate the transfer of mobile genetic elements (MGEs) among bacteria, whereas effector translocators deliver protein effectors to eukaryotic target cells to aid in various infection processes (2, 3). Despite these different functions, recent studies have established that these two large subfamilies share many mechanistic and architectural features (4, 5). In line with these findings, detailed phylogenetic analyses strongly indicate that effector translocators evolved from ancestral conjugation machines during establishment of bacterial pathogenic or symbiotic relationships with eukaryotic hosts (6, 7).

With such an evolutionary trajectory, a central question arises: how were systems dedicated to horizontal transmission of genetic material adapted for translocation of protein effectors, which for some systems number in the hundreds? A partial answer derives from an understanding of the mechanism of conjugative DNA transfer (2). This process is initiated by the binding of a protein termed the relaxase and one or more accessory factors at the origin-of-transfer (*oriT*) sequence of a mobile element. The relaxase catalyzes nicking of the DNA strand destined for transfer (T-strand), and then remains covalently bound to the 5′ end of the T-strand, resulting in a relaxase-T-strand transfer intermediate (T-complex). The relaxase is unfolded by one or more ATPases located at the channel entrance (8), where it then pilots the tethered T-strand through the channel to bacterial recipients. Conjugation machines therefore can be viewed as protein translocation systems that evolved to recognize a specialized class of rolling circle replicases, the relaxases, as substrates, and only coincidentally convey the DNA element to target cells. Indeed, several conjugation systems have been shown to translocate relaxases independently of the co-transferred T-strand, confirming that relaxases are *bona fide* substrates of conjugation machines (8–12).

Effector translocators thus evolved from ancestral conjugation systems through the diversification of protein substrates. Many substrates of effector translocator systems harbor signatures of eukaryotic proteins, including but not limited to Rho- and Rab-GTPase domains and F-box, U-box, and SET domains (13–16). In the most extreme examples of effector diversification, the *Legionella pneumophila* and *Coxiella burnetti* Dot/Icm systems translocate more than a hundred protein effectors during infection, many with eukaryotic-like domains (15, 17–21). Indeed, analyses of 80 *Legionella* genomes spanning 58 species identified genes for more than 18,000 Dot/Icm effectors, showing an astounding capacity of conserved Dot/Icm systems among different *Legionella* species to recruit large numbers of distinct substrate repertoires (16). Such genomic analyses suggest the emergence of T4SS effector repertoires can be accounted for by the acquisition of eukaryotic-like genes or domains into bacterial genomes *via* one or more of the mechanisms (conjugation, transformation, transduction) underlying genetic variation (15, 16).

Although the above explains how T4SS substrate diversity can evolve, the translocated effectors also must carry translocation signals (TSs) required for recognition by T4SSs. Studies have shown, however, that TSs of protein effectors vary considerably between and even within a given system and can consist of C-terminal motifs enriched in positively charged or hydrophobic residues, N-terminal signals of unspecified sequence characteristics, or internal sequence or structural motifs (22–28). Genome-incorporated sequences encoding eukaryotic proteins or domains might have acquired TSs through recombinogenic shuffling with relaxase genes, but it is unlikely that relaxases have supplied the full spectrum of TSs conferring T4SS effector recognition. Prior studies reported evidence for the transfer of a few other plasmid-encoded proteins through conjugation machines, including Sog primase, ParM partitioning protein, and VbhT FIC toxin (29–33). Recently, we expanded the list of protein substrates of the IncFV plasmid pED208 conjugation system to include not only the TraI relaxase, but additionally 16 other proteins encoded by genes clustered within the plasmid’s maintenance and leading region (MLR) (34, 35). We further discovered that the translocation of two such substrates, PsiB and SSB, suppresses the SOS response in new transconjugants, which is activated by sensing the incoming ssDNA transfer intermediate (34).

In this study, we tested the overarching hypothesis that conjugation systems translocate many proteins between bacteria. We developed a high-throughput screen based on the Cre Recombinase Assay for Translocation (CRAfT) to identify the entire repertoire of protein substrates of the pED208-encoded T4SS (hereafter T4SS_ED_) encoded by the *Escherichia coli* W3110 chromosome. We identified 32 chromosomal substrates, all of which have known or predicted functions in DNA metabolism, stress mitigation, transcriptional regulation, or toxin antagonism. Based on these functions, we propose that the co-transfer of proteins along with the pED208 transfer intermediate enhances plasmid reestablishment by mitigating transient metabolic burdens and other stresses accompanying bacterial mating. We further determined that the chromosomally encoded substrates have C-terminal or internal TSs that can act independently or synergistically and that many of these substrates also are translocated through heterologous conjugation systems encoded by the IncN pKM101 and IncP RP4 plasmids. Together, our findings underscore the highly promiscuous nature of conjugation systems in interbacterial trafficking of DNA and protein macromolecules and suggest new scenarios by which the T4SSs evolved their repertoires of specialized substrates.

## RESULTS

### Fusion of Cre to the ASKA ORFs library

**Fig 1:**
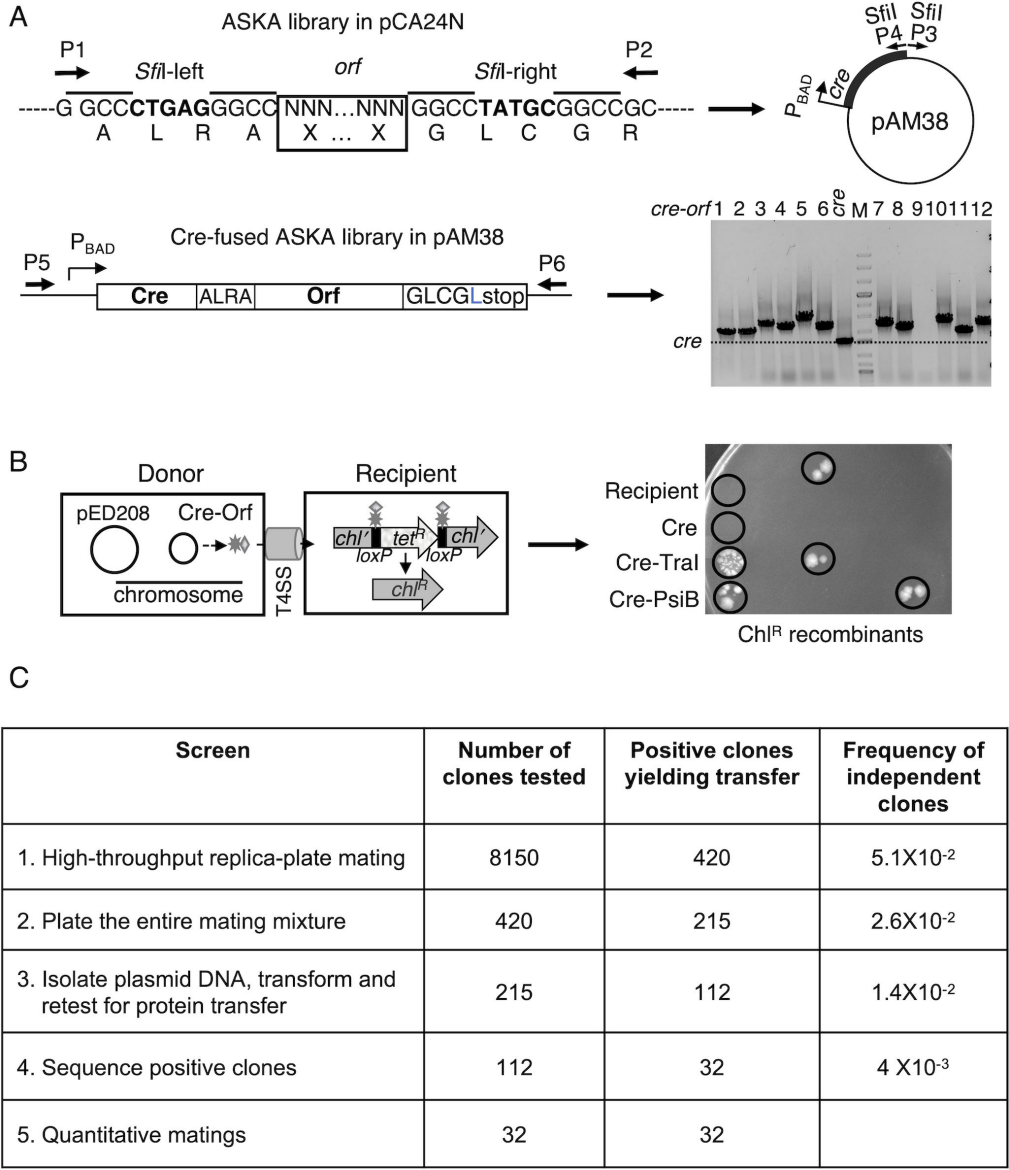
Identification of chromosomally encoded protein substrates of the pED208 conjugation system. (A) Schematic of *E. coli* ASKA library in pCA24N. The cloned *orf* begins at codon #2 and ends at the last codon. It is flanked by noncompatible *Sfi*I sites (noncompatible sequence shown in bold font), resulting in directional cloning. Primers P1 and P2 were used to amplify the *orfs* in the library (in P2, one nucleotide was modified to replace positively charged arginine (**R**) with leucine (**L**)). pAM38 with P_BAD_::*cre* was amplified with primers P3 and P4, respectively, containing *Sfi*I-left and *Sfi*I-right sites, and the *Sfi*I-digested amplicons were ligated to the amplified *orf* library. Primers P5 and P6 were used to verify inserts and to obtain sequences of cloned *orfs*. The gel shows amplification products for 12 *cre-orf* fusions carried by donors mediating Chl^R^ recombinants in the high-throughput CRAfT. (**B)** Schematic: MC4100(pED208) strains carrying the library of Cre-ASKA plasmids served as donors for detection of Cre transfer to CSH26Cm:: LTL, which carries an excisable *loxP* cassette. Transfer of Cre fusion proteins and *loxP* cassette excision results in the growth of colonies with Chl^R^, Tet^S^ recombinant phenotype. Right: A replica plate resulting from the high-throughput screen, with circles highlighting Recipient-only and Cre-only negative controls, results from matings with pED208-carrying donors producing Cre-TraI or Cre-PsiB (positive controls), and results of three matings in which donors presumptively transferred Cre-Orfs mediating excision of the *loxP* cassette in recipients. (**C) **Frequencies of positive clones through the successive screens.

To identify the collection of *E. coli* proteins translocated through the pED208-encoded T4SS, we fused *cre* recombinase to the *E. coli* W1130 ASKA library of genes ranging in sizes from 100 bps to 2.5 kb (Fig. 1A). We introduced the *cre-orf* expression plasmids *en masse* into MC4100(pED208), and tested the capacity of 8,150 resulting strains for conjugative transfer of Cre-Orf fusion proteins to the CSH26Cm::LTL reporter strain using a high-throughput version of CRAfT (Fig. 1B). In this assay, Orf proteins carrying translocation signals recognizable by a given T4SS will mediate transfer of the Cre-Orf fusion protein to target cells, whereupon Cre binds and excises *loxP* cassettes engineered into target cell chromosomes, resulting in a scorable phenotype. Here, we assayed for Cre-mediated excision of a *loxP-tet^R^-loxP* cassette inserted within a *chl^R^* gene, as evidenced by the growth of Chl^R^ recombinant colonies exhibiting Tet^S^. We previously showed that in 20-h solid-surface matings, MC4100(pED208) producing Cre from the *cre*-only vector plasmid fails to translocate Cre, whereas isogenic donors transfer the Cre-PsiB or Cre-TraI fusion proteins at frequencies of ~10^−3^ to 10^−4^ recombinants/donor (Rcs/D) (34, 35). In the nonquantitative liquid matings deployed in the initial high-throughput screen, donors similarly failed to translocate Cre at detectable frequencies, and translocation of Cre-PsiB and Cre-TraI routinely yielded between 5 and 20 recombinant colonies (Fig. 1B).

From our initial screen, we identified 420 strains that yielded two or more *loxP* recombinant colonies, representing a frequency of 5.1 × 10^−2^ positive clones (Fig. 1B and C). We repeated the high-throughput mating protocol with these 420 strains as donors but plated the entire mating mix on selective plates, which resulted in the identification of 215 strains yielding >10 recombinant colonies. Plasmids were isolated from the 215 strains and reintroduced into *E. coli* MC4100(pED208) for a third round of matings, resulting in 112 donor strains that reproducibly yielded at least 10 recombinant colonies in 20-h liquid matings. Plasmids recovered from these donors were sequenced across the entire *cre-orf* fusion, resulting in the identification of 32 unique genes, of which all were in-frame fusions and many were identified multiple times (Fig. 1C; Table 1). Based on the number of strains tested for protein transfer and the number of multiple hits for the 32 genes, we estimate that the ASKA library was screened at 97% saturation as per calculations presented in reference (36).

**TABLE 1:** Features of chromosomally encoded proteins delivered through the T4SS_ED_

Name	Accession number	AAs/size kDa Location	Known or predicted function	# Hits^*a*^	References
Group 1 DNA/nucleotide metabolism					
DinI	BAA06515.1	81/8.95 cytoplasm	DNA damage is inducible. Concentration-dependent stabilization or depolymerization of RecA-ssDNA filament.	1	(37–39)
FrlC	BAE77918.1	276/31.1 cytoplasm	Fructoselysine 3-epimerase. Possesses a conserved domain of AP endonuclease family 2	1	(40, 41)
NrdE	BAA16539.2	714/80.5 cytoplasm	Class 1b Ribonucleotide reductase subunit alpha. RNRs catalyze the conversion of nucleotides to deoxynucleotides, providing the monomeric building blocks required for DNA replication and repair	4	(42, 43)
NudI	APC52490.1	140/16.3 cytoplasm	Pyrimidine deoxynucleoside triphosphate pyrophosphohydrolase. Hydrolyzes dUTP to dUMP +PP	4	(44, 45)
TatD	BAE77462.1	260/29.0 cytoplasm	3'-5' exonuclease with a preference for single strand DNA and RNA	14	(46)
YhcC	BAE77255.1	309/34.6 cytoplasm	Catalyzes the reductive cleavage of S-adenosylmethionine to methionine and 5'-deoxyadenosine.	1	(47)
YiaL	BAE77717.1	155/17.6 cytoplasm	Promotes radiation survival. Possible role in sialic acid metabolism and biofilm formation.	1	(48)
YrfG	BAE77892.1	222/25.4 cytoplasm	Purine nucleotidase	20	(49)
YfjX	BAA16511.1	152/17.3 cytoplasm	CP4-57 prophage anti-restriction protein.	1	(50)
Group 2 Stress/physiology					
Can	NP_414668.1	220/25.1 cytoplasm	Carbonic anhydrase 2. Acid tolerance/adaptation.	1	(51)
EntD	BAA35224.2	209/23.6 IM, cytoplasm	Enterobactin synthase component D enterobactin biosynthesis. Metal and oxidative stress tolerance.	3	(52, 53)
Fpr	BAE77386.1	248/27.8 cytoplasm	Flavodoxin/ferredoxin-NADP(+) reductase. Oxidative stress tolerance.	1	(54)
GadA	BAE77777.1	466/52.7 IM, cytoplasm	Glutamate decarboxylase. Acid tolerance.	4	(55)
GpmM	UDE09060.1	514/56.2 cytoplasm	2,3-bisphosphoglycerate-independent phosphoglycerate mutase. Oxidative stress tolerance.	6	(56, 57)
HemG	APC53577.1	181/21.3 IM, cytoplasm	Protoporphyrinogen oxidase, heme b biosynthesis. Protection from oxidative stress by decomposition of hydrogen peroxide.	1	(57, 58)
MiaB	BAE76357.1	474/53.7 cytoplasm	tRNA-N (6)-(isopentenyl)adenosine-37 thiotransferase. Possible role in oxidative stress sensing.	3	(59, 60)
RhaD	BAE77407.1	274/30.2 cytoplasm	Rhamnulose-1-phosphate aldolase.	2	(61)
RspA	BAA15285.1	404/46.0 cytoplasm	D-altronate/D-mannonate dehydratase. Regulatory role in starvation sensing through autoinducer signaling.	3	(61)
RspB	BAA15284.1	339/36.6 cytoplasm	Putative zinc-binding oxidoreductase. Possible role in oxidative stress tolerance.	2	(61)
SodA	BAE77401.1	206/23.1 cytoplasm	Superoxide dismutase. Oxidative stress tolerance.	1	(62)
SpeE	BAB96695.1	288/32.3 cytoplasm	Spermidine synthase. Protection from oxidative stress through induction of *oxyR* and *rpoS* gene expression	1	(63, 64)
YciE	BAA14789.1	168/19.0 cytoplasm	Ferriten-like metal-binding protein. Induced under osmotic stress.	1	(65)
YdhF	BAE76490.1	298/33.7 cytoplasm	Oxidoreductase. Oxidative stress tolerance.	3	(66)
YihT	BAE77428.1	292/32.0 cytoplasm	6-deoxy-6-sulfofructose-1-phosphate aldolase, a part of the sulfoquinovose degradation I pathway. Adaptation to phosphate stress.	1	(67, 68)
YihV	BAE77426.1	298/31.7 cytoplasm	6-Deoxy-6-sulfofructose kinase. Part of the sulfoquinovose degradation I pathway. Adaptation to phosphate stress.	1	(67, 68)
YphB	BAE76730.1	290/32.7 cytoplasm	Aldose 1-epimerase. Part of the sulfoquinovose degradation I pathway. Adaptation to phosphate stress.	3	(68, 69)
Group 3 Transcription regulators					
RfaH	BAE77461.1	162/18.3 cytoplasm	Transcriptional antiterminator. Enhances *tra* operon expression.	3	(70)
YbeF	BAA35272.1	317/36 cytoplasm	LysR-type DNA-binding transcriptional regulator. Regulates flagella production.	12	(71, 72)
Group 4 Antitoxins					
CbeA	WP_001285584.1	122/13.7 cytoplasm	CP4-44 prophage. Cytoskeleton bundling-enhancing antitoxin of CbtA-CbeA TA system.	1	(50)
TabA (YjgK)	APC54376.1	150/16.9 cytoplasm	Putative anti-toxin. DUF386 domain-containing TA biofilm protein.	1	(73)
YagB	BAE76052.1	116/13.2 cytoplasm	Putative anti-toxin. CbeA/YafW/YfjZ family.	1	(50)
YfjZ	BAA16513.1	105/11.7 cytoplasm	CP4-57 prophage. Putative antitoxin of YpjF-YfjZ TA system.	1	(50)

^*a*^ Number of times the gene was identified in our high-throughput screen.

**Fig 2:**
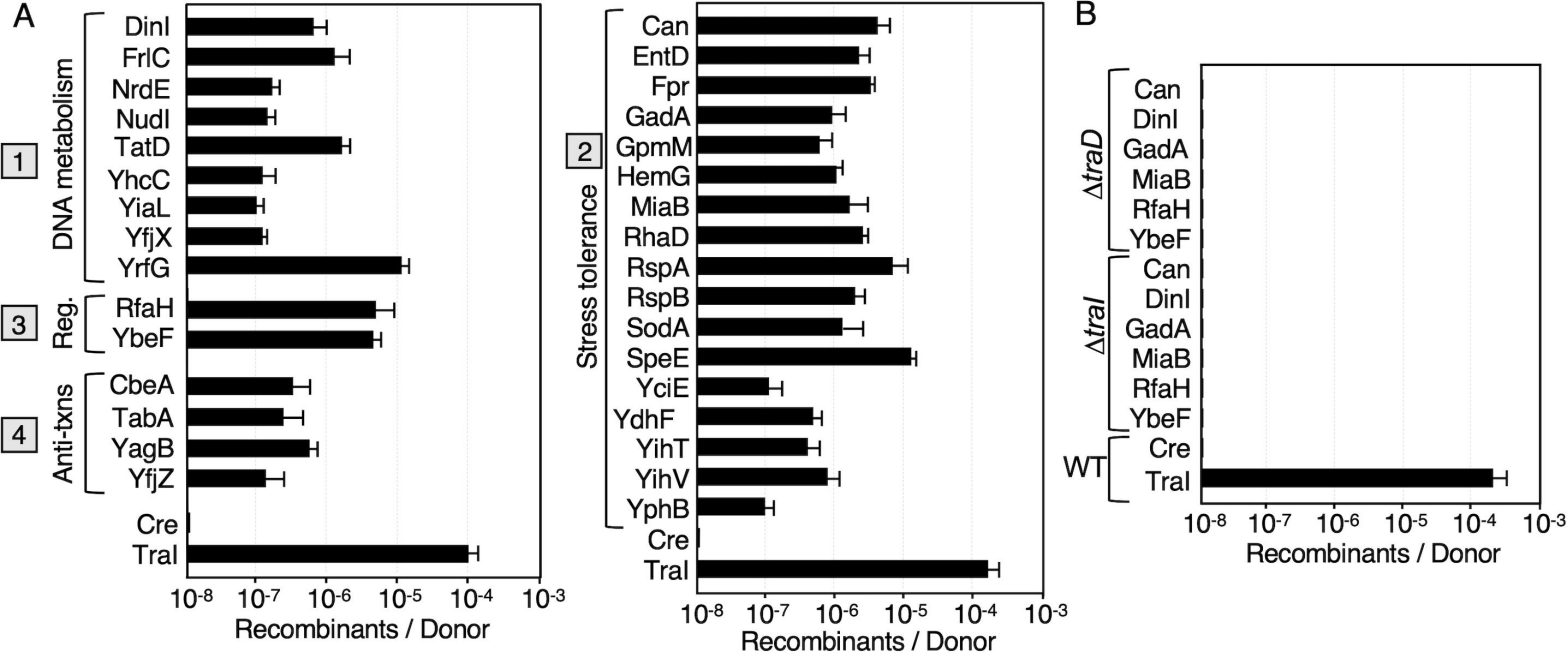
Transfer frequencies of Cre fusion proteins through the T4SS_ED_, as monitored by CRAfT. (A) Transfer of Cre fused to the proteins listed; proteins were assigned Groups 1 to 4 according to known or predicted functions. MC4100(pED208) donors harboring plasmids encoding the respective Cre-Orf fusion proteins were mated with the CRAfT reporter strain CSH26Cm:: LTL. (**B)** Results of matings with MC4100 donors carrying pED208Δ*traD* or pED208Δ*traI* mutant plasmids and producing Cre fused to the proteins shown. Cre transfer frequencies are reported as the number of Recombinants per Donor. Experiments were repeated at least three times in triplicate, and results from a representative experiment with standard errors are shown.

In quantitative liquid mating assays, pED208-carrying donors delivered the 32 Cre-Orf fusion proteins at frequencies from ~10^−7^ to nearly 10^−5^ recombinants per donor (Rcs/D) (Fig. 2A). Although lower than observed for Cre-TraI or Cre-PsiB transfer, these transfer frequencies are comparable to those reported previously for transfer of Cre fused to the pED208-encoded MLR proteins (34, 35). A donor strain deleted of the TraD T4CP, which functions as the substrate receptor for this T4SS, failed to translocate the Cre-Orf fusion proteins, confirming the essentiality of a functional T4SS_ED_ for protein transfer (Fig. 2B). Moreover, as shown previously for the Cre-MLR fusion proteins (34, 35), donors deleted of the TraI relaxase failed to translocate the tested Cre-Orf fusions (Fig. 2B). Docking of TraI and, presumably, the pED208 plasmid substrate, with the transfer channel thus appears to be a general requirement for protein transfer through the T4SS_ED_ (34, 35). We screened at least 25 recombinant colonies arising from the transfer of each of the 32 Cre-Orf fusion proteins, and invariably all had acquired pED208 as evidenced by a *Spc^R^* phenotype. These findings suggest that DNA substrate docking with the T4SS_ED_ might serve to activate the T4SS_ED_ and ensure co-translocation of the plasmid and protein substrates into the same recipient cells.

### Features of the translocated proteins

The 32 proteins identified by high-throughput CRAfT are known or predicted to localize in the bacterial cytoplasm, as expected of T4SS substrates (Table 1). They range in size from ~9 to 81 kilodaltons (kDa). Nearly all have known or predicted functions that could aid in plasmid reestablishment or mitigation of stresses associated with plasmid acquisition. For example, nine substrates (designated as Group 1 substrates) have known or predicted effects on DNA or nucleotide metabolism (Table 1). One of these, DinI, potentially modulates the SOS response (37–39), which we and others have shown is activated in new transconjugants upon uptake of the ssDNA transfer intermediate (34, 74, 75). Another translocated protein, YfjX, has a predicted anti-restriction function that might protect the incoming T-strand from degradation in new transconjugants. Other translocated proteins in this group include FrlC (fructoselysine 3-epimerase), NrdE and NudI (pyrimidine deoxynucleoside triphosphate pyrophosphohydrolase), TatD (3′ - 5′ exonuclease with preference for ssDNA and RNA), YhcC (reductive cleavage S-adenosylmethione to methionine and 5′-deoxyadenosine), YrfG (purine nucleotidase), and YiaL (possible role in DNA repair in response to UV- or X-radiation). These translocated functions might serve to counteract metabolic perturbations accompanying the acquisition of the F plasmid in new transconjugant cells, possibly by aiding in the growth of transconjugants through the generation of DNA precursors or metabolic signals or mitigating the SOS response or other mating-induced stresses.

In all, 17 translocated proteins are known or predicted to confer tolerance to environmental stresses, including oxidative stress (EntD, Fpr, GpmM, HemG, SpeE, SodA, and YdhF), phosphate stress (YihT, YihV, and YphB), osmotic stress (YciE), acidic conditions (Can and GadA), and starvation (RspA and RspB) (Table 1). We also provisionally assign MiaB (Isopentenyl-adenosine A37 tRNA methylthiolase) and RhaD (Rhamnulose-1-phosphate aldolase) to this group for their possible indirect roles in stress adaptation or signaling. Translocation of the Group 2 proteins might serve to counteract various stress response pathways activated by mating and F plasmid acquisition.

Two translocated proteins, RfaH and YbeF, are transcriptional regulators (Table 1). RfaH is of particular interest in view of previous work showing that it is a transcriptional antiterminator required for the expression of the F plasmid *tra* operon (70, 76, 77). Translocation of RfaH along with the DNA substrate might aid in the proliferation of the F plasmid through zygotic induction, a phenomenon involving transient derepression of *tra* gene expression and a burst of F plasmid propagation by new transconjugants (78, 79). The second transcriptional regulator, YbeF, is a LysR-type DNA-binding protein recently shown to control the expression of *fhlDC,* which encodes the master regulator for flagellar biogenesis, as well as genes involved in oxidative phosphorylation and other biomolecular processes (71, 80). Translocation of YbeF might serve to downregulate flagella production to conserve energy needed for F plasmid regeneration/maintenance or regulate processes enabling cells to cope with mating-induced stresses.

Finally, three translocated proteins, CbeA, YagB, and YfJZ, are antitoxins characteristically encoded within prophages (Table 1). This finding is of interest given that RecA and the SOS response are known inducers of prophage lytic programs and toxin-antitoxin systems (81, 82). Conceivably, translocation of these antitoxins serves to block phage-encoded or other toxins produced as a component of the mating-induced SOS response. A fourth protein, TabA (Toxin-antitoxin biofilm protein), is included in this group for its role in controlling biofilm development and dispersal as well as fimbriae production in response to the perception of autolytic activities associated with five toxin components of TA systems encoded within the *E. coli* chromosome (73). Translocation of TabA might serve to block the expression of TA systems and autolysis of new transconjugants in response to mating-induced stress.

### Effects of gene deletions in donors and recipients on pED208 transfer

To determine whether protein translocation impacts the efficiency with which pED208 is successfully transmitted to recipient cells, we introduced pED208 into strains deleted of the genes of interest from the Keio collection (83). The Keio collection lacks strains deleted of *hemG, entD, rhaD,* or *can*, presumably because their products are essential for cell viability. We therefore screened 28 mutant donors for pED208 transfer and identified only one mutation (*rfaH*) that significantly attenuated plasmid transfer, yielding a transfer frequency of 10^3^-fold lower than observed for MC4100(pED208) donors (Fig. S1A). As noted above, *rfaH* encodes an antiterminator, and in early studies was shown to significantly enhance the efficiency of transcription through the long ~30 kb *tra* operon (76, 77). We therefore attribute the reduced capacity of the Δ*rfaH* mutant to transfer pED208 to attenuated transcription of the pED208 *tra* operon and a reduction in T4SS_ED_ channel and ED208 pilus production. In line with this proposal, MC4100(pED208) cells are highly aggregative because of abundant production of ED208 pili (84), but Δ*rfaH*(pED208) variants were strongly attenuated in this autoaggregation phenotype (Fig. S1A).

The lack of detectable effects of protein translocation on pED208 transfer could be due to the use of MC4100 as the recipient strain, which is competent for the production of the translocated proteins. We, therefore, tested whether strains deleted of genes for the translocated proteins serve as poor recipients for pED208. MC4100(pED208) donors were mated with deletion mutants from the Keio collection, but invariably pED208-carrying donors efficiently translocated the plasmid to all mutant strains (Fig. S1B). Except for the importance of RfaH production in donor cells, the chromosomally encoded proteins thus appear not to detectably impact pED208 transfer when produced in donor or recipient cells.

### Deletions of genes whose products are involved in DNA/nucleotide metabolism trigger the SOS response in donor populations

Recently, by single-cell imaging and flow cytometry, we confirmed early reports that conjugation induces the SOS response in recipient cells through the transfer of the ssDNA intermediate, which serves as an inducing signal for this stress response (see Fig. S2A) (34, 74, 75). In this assay, donor strains are mated with a recipient strain carrying the SOS-responsive reporter P*_sulA_-mCherry* in its chromosome, and the mating mix is monitored by flow cytometry for an increase in the numbers of cells with mCherry fluorescence at levels above a “red” gate, which is set with the SOS induction-deficient *lexA3* reporter strain (see Fig. S2B and C) (34, 85, 86). Prior work has established that the emission wavelength, rapid expression, and long half-life of mCherry allow for sensitive detection of living cells in which SOS has been induced by flow cytometry (71, 81).

Using this assay, we showed that MC4100(pED208) donors elicit a significant increase in the number of recipient reporter cells exhibiting an SOS response as compared to plasmid-free “donors” (34). We further showed that when compared with pED208-carrying donors, donors with pED208 variants deleted of *psiB* or *ssb* trigger a significantly stronger SOS response in new transconjugant cells. Conversely, donors competent for translocation of the PsiB or SSB proteins during mating mitigate the mating-induced SOS response in new transconjugants. Conjugative DNA transfer thus elicits the SOS response, whereas translocation of PsiB and SSB suppresses the magnitude of this stress response in new transconjugants. Based on these findings, we proposed that conjugative protein transfer can serve to mitigate potentially deleterious consequences to plasmid maintenance and propagation that accompany SOS activation, for example, error-prone replication (34).

Here, we asked whether donor cells lacking genes for the Group 1 cohort of protein substrates identified above (Table 1; DNA/nucleotide metabolism) elicit heightened SOS responses when mated with the SOS reporter strain, as observed previously for the Δ*psiB* or Δ*ssb* mutant donors. To this end, we generated a set of MC4100(pED208) strains deleted of *dinI*, *frlC*, *nrdE*, *nudI*, *tatD*, *yhcC*, *yiaL*, *yrfG*, or *yfjX* by P1 transduction. The resulting donors were isogenic with the previously characterized MC4100 strains harboring pED208, pED208Δ*psiB,* or pED208Δ*ssb*. Upon mixing the mutant donors with the P*_sulA_-mCherry* reporter strain, however, we observed no appreciable differences in SOS responses compared with the response elicited by MC4100(pED208) (Fig. S2B and C).

**Fig 3:**
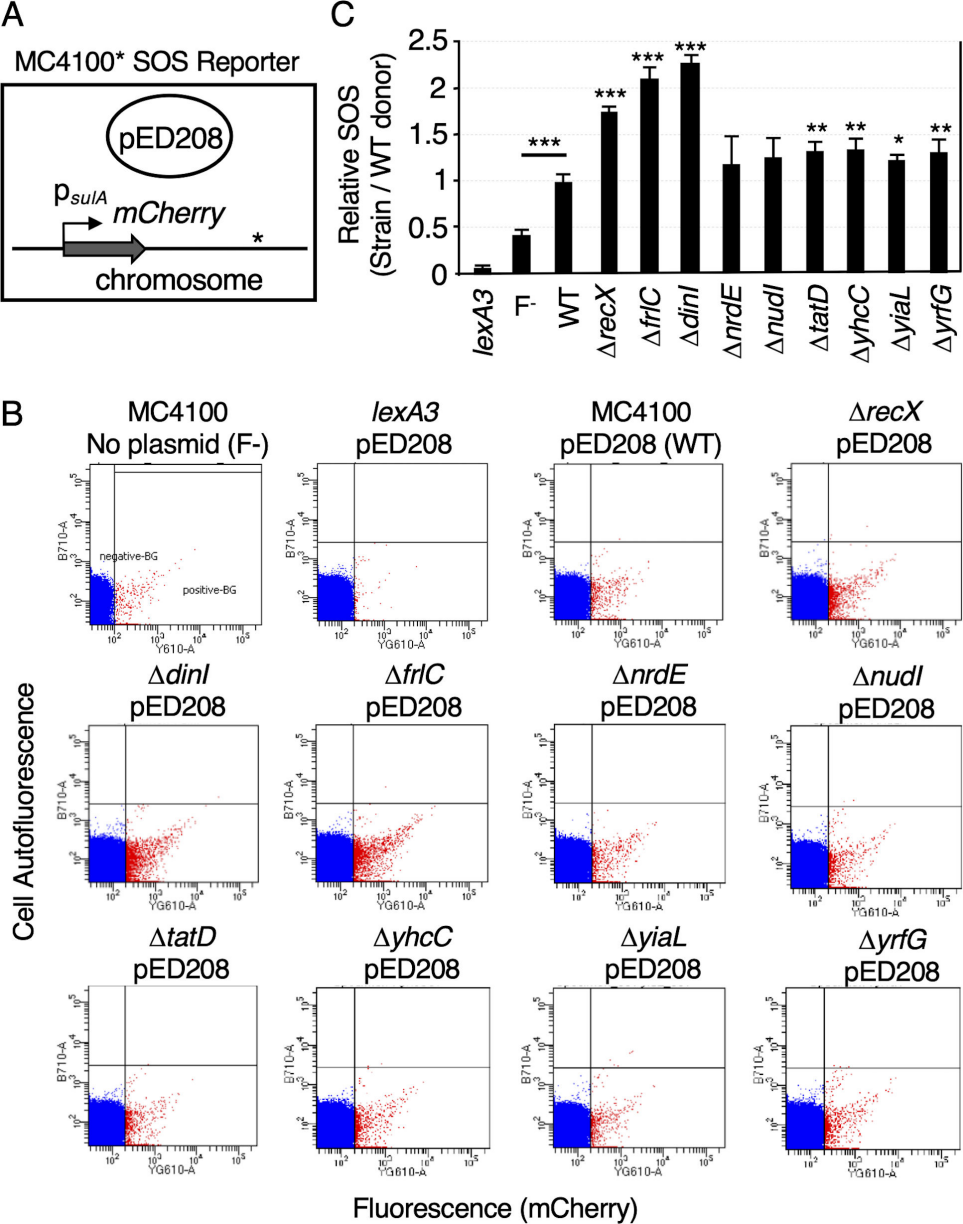
Deletions of DNA metabolism genes enhance the SOS response elicited by the carriage of the F plasmid. (**A)** Schematic of MC4100 mutant strains with the SOS reporter (P*_sulA_*-mCherry) and carrying IncFV pED208. The asterisk denotes chromosomal deletions of the Group 1 genes with known or predicted functions in DNA/nucleotide metabolism. (**B)** Representative examples of flow cytometry data for MC4100 strains carrying the P*_sulA_*-mCherry SOS reporter. Upper panels show MC4100 alone or carrying pED208 (WT), the SOS-uninducible strain *lexA3*(pED208), or the Δ*recX*(pED208) mutant, which exhibits a high-level SOS response. Lower panels show SOS induction levels by pED208-carrying MC4100 with deletion mutations shown. Data points indicate flow cytometry events (cells) colored red to the right (SOS induced) and blue to the left (SOS uninduced) of the “red” gate, which was set using the SOS-uninducible *lexA3* mutant strain harboring pED208. X- and Y-axes denote mCherry fluorescence and cell autofluorescence, respectively. (**C)** Quantitation of the relative SOS responses exhibited by strains depicted in panel B. Results are presented as the relative SOS response, which corresponds to the ratio between the numbers of mutant cells vs MC4100(pED208) cells (set to 1) exhibiting SOS induction. Values are means plus standard errors of means (SEM) (error bars). *P* values were determined using the two-tailed Student’s *t*-test: *P < 0.05; **P < 0.005; ***P < 0.0005. The *P* value for MC4100(pED208) (denoted WT) vs plasmid-free MC4100 (F-) is underlined; *P* values above each bar denote comparisons between each pED208-carrying mutant strain and MC4100(pED208).

We previously reported that MC4100(pED208) cells exhibit a ~ 1.7-fold higher level of SOS activation than observed for plasmid-free MC4100 (34). Furthermore, MC4100(pED208) mutants deleted the genes required for plasmid transfer, for example, *traA* pilin, *traD* substrate receptor, *traI* relaxase, exhibited SOS levels resembling that of the plasmid-free strain. These findings suggested that the elevated SOS response exhibited by pED208-carrying cells is attributable mainly to redundant pED208 transfer in the donor cell population (34). In view of these findings, we asked whether MC4100*(pED208) cells deleted the genes for chromosomal substrates of the T4SS_ED_ and carrying the P*_sulA_-mCherry* reporter construct show an altered SOS response compared to WT MC4100(pED208) cells (Fig. 3A).

Reminiscent of our earlier findings, MC4100(pED208) cells exhibited a significantly higher SOS response than plasmid-free MC4100, whereas the SOS nonactivatable *lexA3* mutant showed only background SOS levels (Fig. 3B and C). As expected, the deletion of *recX*, a known inhibitor of RecA filament formation (39, 87), exacerbated the SOS response, resulting in a ~ 1.7-fold increase in the relative SOS response over that observed for MC4100(pED208) donors. Strikingly, the Δ*dinI* and Δ*frlC* mutations conferred SOS responses comparable to those elicited by the Δ*recX* mutation (Fig. 3B and C). The Δ*tatD,* Δ*yhcC,* Δ*yiaL,* and Δ*yrfG* mutations also elevated the SOS response by statistically significant levels. Production of DinI, FrlC, TatD, YrcC, YiaL, and YrfG thus appears to directly or indirectly counteract the stimulatory effects of pED208 carriage and redundant transfer on SOS induction.

### Translocation signals of the T4SS_F_ substrates

**Fig 4:**
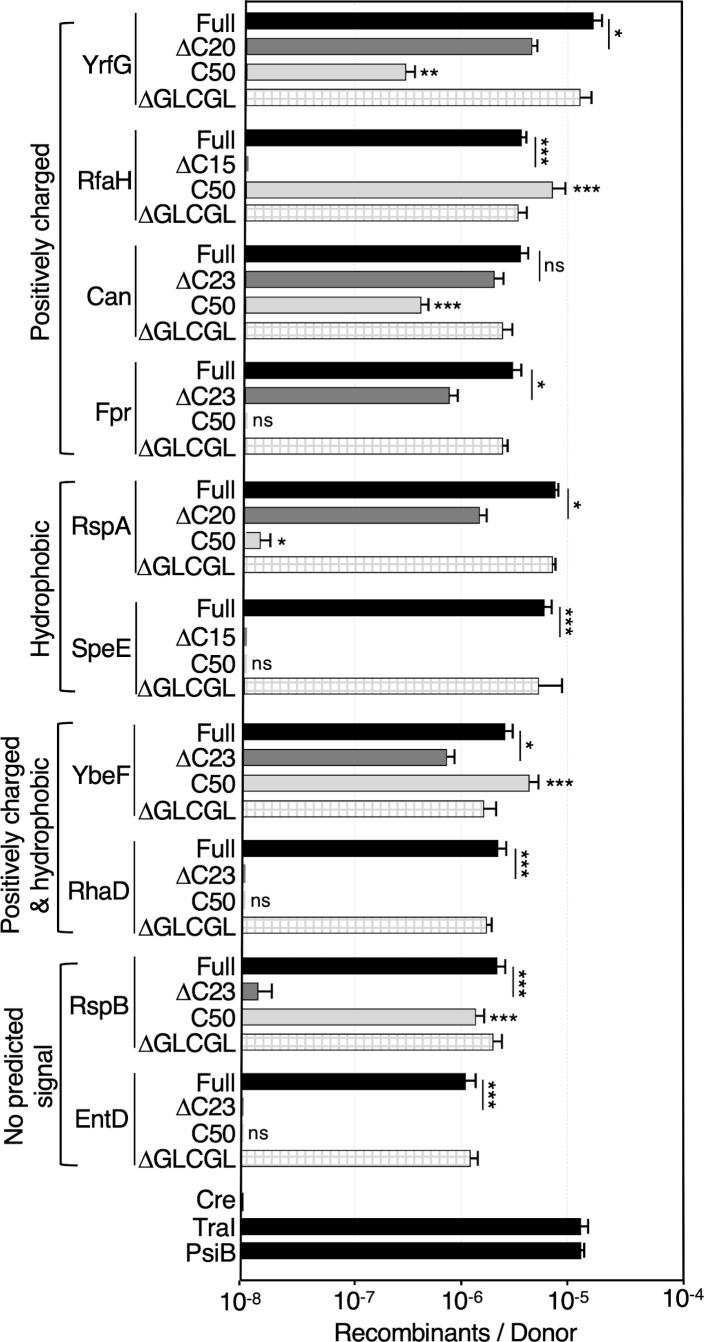
Contributions of C-terminal residues to protein transfer. Transfer of Cre fused to C-terminal truncations or cloned C termini of proteins listed. Proteins are grouped according to the charge and hydrophobicity characteristics of their C-terminal 25 residues (Table S2). MC4100(pED208) donors harbored plasmids encoding Cre-Orf truncations (denoted ΔXX, where XX corresponds to the number of residues deleted), Cre fused to the C-terminal 50 residues (**C50**) of the corresponding protein, or Cre-Orf fusions deleted of the GLCGL motif resulting from *en masse* cloning of the ASKA library by *Sfi*I digestion (see Fig. 1A). Transfer of the Cre fusion proteins to the Cre reporter strain CSH26Cm:: LTL are reported as the number of Recombinants per Donor. Experiments were repeated at least three times in triplicate, and results from a representative experiment with standard errors are shown. *P* values determined by Student’s *t*-test are presented for comparisons between the full-length protein vs corresponding C-terminal truncation mutant (underlined), or Cre fused to C-terminal 50-residue fragment vs Cre only: *P < 0.05; **P < 0.005; ***P < 0.0005; ns, not significant.

Translocation signals (TSs) conferring recognition of protein substrates by effector translocators can be located C-terminally or internally, and some effectors carry two or more internal and terminal TSs (22–28). We initiated studies of TSs borne conferring recognition of the chromosomally encoded substrates of the F system by testing whether C-terminal residues are required for transfer. We used the algorithm https://www.bachem.com/knowledge-center/peptide-calculator/ to identify the overall charge and hydrophilic characters of the last 25 residues, which resulted in assignment of the 32 substrates into one of four groups: (i) net positive charge (net charge >1, hydrophilicity ≥−0.1), (ii) hydrophobic (net charge <1, hydrophilicity <−0.1), (iii) net positive charge and hydrophobic (net charge >1, hydrophilicity <−0.1), and (iv) no predicted signal (net charge <1, hydrophilicity >−0.1) (Table S2). We selected 10 substrates representative of the four groups to assess the effects of C-terminal truncations (ΔCT) and of cloned C-terminal residues (C50) on Cre transfer (Fig. 4).

Among the four tested proteins with overall positively-charged C termini (Group 1), the ΔCT mutations had no significant effect on translocation of Cre fused to Can, modestly impacted the YrfG and Fpr fusions, and completely abolished translocation of the RfaH fusion (Fig. 4). The cloned C50 of RfaH supported efficient Cre transfer, strongly indicating that RfaH’s TS resides entirely in its C terminus. The C50s of YrfG and Can also supported Cre transfer, suggesting that these proteins have a C-terminal TS as well as one or more internal TSs and that both types of TSs can function independently of each other. The C50 of Fpr failed to support Cre transfer, suggesting that Fpr’s TS resides internally.

The ΔCTs of substrates in groups 2 to 4 also conferred a range of effects from mild attenuation (RspA and YbeF) to near or complete loss (SpeE, RhaD, RspB, and EntD) of translocation (Fig. 4). The cloned C50s supported (YbeF and RspB) or failed to support (RspA, SpeE, RhaD, and EntD) translocation. We infer from these data that the TS(s) for RspA resides predominantly internally, whereas that of RspB is C-terminal. YbeF has at least two TSs, located internally and C-terminally, that can function independently of each other. Given that neither the ΔCTs nor C50s of SpeE, RhaD, and EntD supported Cre transfer, we propose that the TSs harbored by these substrates span the junctions of the truncations or cloned regions, or they are properly displayed only in the context of the fully intact protein.

Finally, we note that the strategy deployed for cloning of the ASKA *orf* library downstream of *cre* resulted in the addition of five residues (GLCGL) to the C termini of all Cre-Orf fusions (see Fig. 1A). Although these residues were not present among the Cre-ΔCT or Cre-C50 constructs analyzed above, we nevertheless compared translocation efficiencies of the original Cre-Orf fusions with derivatives deleted of the GLCGL motif for 10 substrates. Deletion of this motif did not alter translocation efficiencies (Fig. 4), indicating that this motif does not contribute positively or negatively to their transfer through the T4SS_ED_. We acknowledge, however, that the presence of this motif might have impeded the detectable transfer of some chromosomal substrates of the T4SS_ED_ in our initial high-throughput CRAfT screen.

### *E. coli* proteins are also translocated through the pKM101- and RP4-encoded T4SSs

**Fig 5:**
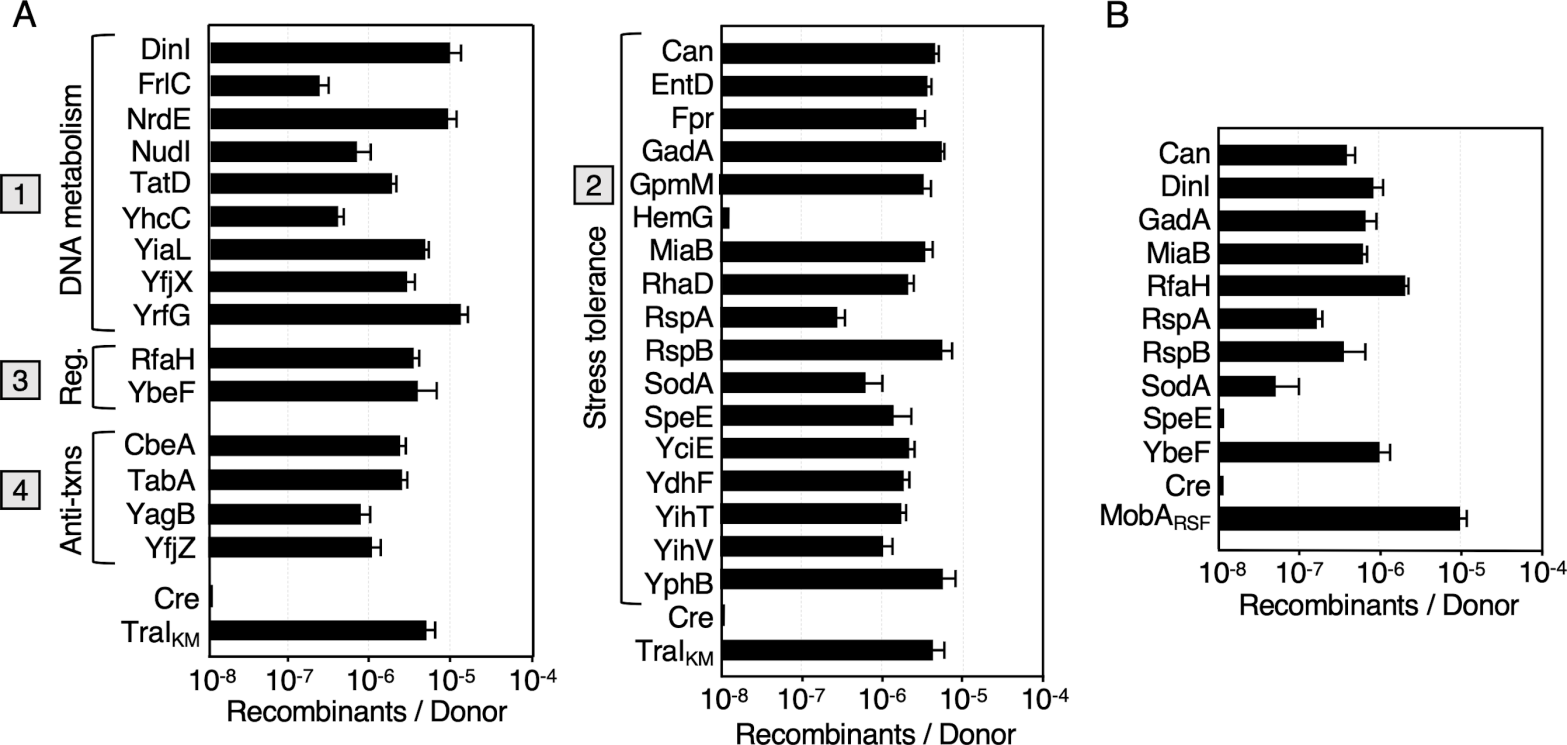
Promiscuous transfer of Cre fusion proteins through the pKM101 and RP4 conjugation systems. (**A)** Transfer of Cre fused to the proteins listed through the pKM101-encoded T4SS (T4SS_KM_); proteins were assigned Groups 1 to 4 according to known or predicted functions. (**B) **Transfer of Cre fusion proteins through the RP4-encoded T4SS (T4SS_RP4_); the RP4 *tra* region is integrated into the chromosome of strain S17-1. As positive controls for protein transfer, Cre was fused to pKM101-encoded TraI relaxase to monitor transfer through the T4SS_KM_ and RSF1010-encoded MobA relaxase for transfer through the T4SS_RP4_. Cre transfer frequencies are reported as the number of Recombinants per Donor. Experiments were repeated at least three times in triplicate, and results from a representative experiment with standard errors are shown.

Recently, we reported that many substrates of the T4SS_ED_ encoded by pED208 promiscuously transfer through other conjugation systems encoded by the IncN plasmid pKM101 and the IncP plasmid RP4 (35). Remarkably, here we found that this was also the case for the chromosomally encoded substrates (Fig. 5A). As a positive control for transfer through the T4SS_KM_, we monitored the translocation of Cre fused to pKM101-encoded TraI relaxase (35). All but one chromosomal substrate, HemG, of the T4SS_ED_ mediated Cre transfer through the T4SS_KM_ (Fig. 5A). Interestingly, most fusion proteins were delivered through the T4SS_KM_ more efficiently than through the T4SS_ED_, with transfer frequencies generally of 10^−6^ - 10^−5^ Rcs/D. Only RspA, SodA, SpeE, and FrlC supported Cre transfer more efficiently through the T4SS_ED_ than the T4SS_KM_ (Fig. 2A and 5A). We also tested a subset of 10 Cre fusion proteins for transfer through the T4SS_RP4_ elaborated by strain S17-1, which carries the RP4 *tra* operon in its chromosome (Fig. 5B). Except for Cre-SpeE, all fusion proteins were transferred through the T4SS_RP4_ channel, at frequencies comparable to those observed for transfer through the T4SS_ED_.

## DISCUSSION

The 32 chromosomally encoded protein substrates of the pED208 conjugation system, together with the 17 plasmid-encoded substrates we recently identified (34, 35), bring the total number of protein substrates of the F system “conjutome” to nearly 50. Of these substrates, many have known or predicted functions associated with DNA metabolism or stress mitigation, suggestive of roles in promoting the establishment of the co-transferred F plasmid in new transconjugants. Recent studies have distinguished transient short-term acquisition costs from longer-term fitness costs associated with the transmission and maintenance of MGEs (75, 88–93). Conjugative DNA transfer of plasmids, for example, introduces transient metabolic perturbations and other stresses in new transconjugant cells through the need to reallocate resources for replication and reestablishment of the acquired MGE (88, 89). These acquisition costs can significantly impact various physiological processes in new transconjugant cells, as evidenced by reductions in growth rates and/or prolonged lag times (88, 89). In view of these findings, and the predicted transient effects of the conjutome substrates upon translocation, we propose that the conjugative protein transfer through F system evolved to mitigate various short-term acquisition costs and stresses experienced by new transconjugant cells.

In support of this model, we previously reported that the translocation of two proteins, PsiB and SSB, which are commonly encoded by F plasmids and many other conjugative plasmids (94–96), suppress the SOS response in new transconjugant cells (34). In these cells, the SOS response is transiently activated upon sensing the incoming ssDNA transfer intermediate. By use of a SOS reporter strain harboring P*_sulA_*-mCherry and single-cell imaging coupled with FACS, we detected stimulatory effects of Δ*psiB* and Δ*ssb* deletion mutations and, conversely, suppressive effects of PsiB and SSB protein translocation on the mating-induced SOS response (34). Here, we extended this line of investigation by testing whether Group 1 substrates also modulate this SOS response. We were particularly interested in DinB because, like PsiB, DinI binds RecA and blocks RecA-ssDNA filamentation. DinI also has been shown to dissolve previously formed RecA filaments (37). However, Din’s role in the SOS response is complicated by the fact that it must be overproduced relative to RecA to suppress this stress response; at stoichiometric levels, DinI confers the oppositive effect of stimulating the SOS response by stabilizing RecA-DNA filaments (38, 39). These stoichiometry-dependent effects on SOS stimulation or suppression might account for our inability to detect effects of the Δ*dinI* mutation on the mating-induced SOS response; conceivably, donor cells do not produce or translocate DinI at sufficient levels to modulate this stress response in new transconjugants, at least under the experimental conditions deployed in our mating assays. We also note that FACS captures only a temporal snapshot of the SOS response exhibited by the reporter strain and that studies with this assay have shown that cells exhibit a highly variable SOS response when exposed to DNA- damaging agents. Even among populations of mutants exhibiting an SOS-constitutive phenotype, for example, *recG*, only a small percentage of cells typically show scorable SOS activation (85, 86, 97). Consistent with these findings, in our mating assays with MC4100(pED208) donors, we detected SOS responses at levels above the “red” gate in only a small fraction (~0.2%) of the total recipient cell population although this fraction corresponds to ~2% to 20% of new transconjugant cells (34). It is conceivable that the SOS reporter system lacked the sensitivity needed to detect small modulatory effects of Δ*dinI* or the other Group 1 deletion mutations on the mating-induced SOS response. These observations underscore the need for further refinements of existing reporter assays as well as the development of new single-cell reporter assays with sufficient sensitivity to identify transient physiological consequences accompanying interbacterial protein transfer.

Interestingly, we also have shown that MC4100(pED208) redundantly transfers the plasmid among the donor population at high frequencies approaching 10^−2^ Tcs/D, and that this population of cells exhibits significantly higher SOS activation than plasmid-free MC4100 (34). Mutations rendering pED208 nontransmissible, for example, Δ*traA*, Δ*traD*, Δ*traI*, attenuate the SOS response nearly to levels observed for MC4100, suggesting that simple carriage and redundant transfer of pED208 imposes a chronic stress that we suggest constitutes a longer-term fitness cost experienced by the donor cell population (34). Although such fitness costs can be associated with metabolic perturbations accompanying reallocation of resources for plasmid replication and maintenance (90–93), expression of the large *tra* operon and assembly of the large envelope-spanning T4SS with its dynamic F pilus also has been shown to elicit extracytoplasmic and cytoplasmic stress responses among F-carrying donor cells (98). Redundant plasmid transfer likely imposes further stresses resulting from competition between resident F and incoming F plasmids. For example, while resident plasmids encode various energetically costly mechanisms such as entry/surface exclusion, anti-restriction, and incompatibility to thwart entry of incoming F plasmids, incoming plasmids capable of bypassing these blockades compete with resident F plasmids for resources required for the establishment and propagation in the new host cell.

The various stresses experienced by donor cells through plasmid maintenance and propagation thus can be distinguished from the transient acquisition costs experienced at the time of plasmid uptake by new transconjugants. This could explain why we detected different modulatory effects of certain conjutome gene deletions on SOS responses exhibited by one but not the other of these cell populations. Although the Δ*psiB* or Δ*ssb* mutations in donor cells elicit significantly higher mating-induced SOS responses in new transconjugant cells, neither mutation detectably impacts the SOS response associated with pED208 carriage (34). By contrast, the Δ*parA* and Δ*parB1* mutations triggered ~2-fold increases in the SOS induction in donor cell populations without impacting the mating-induced SOS response exhibited by new transconjugant cells (34). ParA and ParB1 might suppress the effects of pED208 carriage on the SOS response by ensuring faithful plasmid replication and partitioning of the resident plasmid, and possibly also by blocking the successful establishment of redundantly transferred DNA through partition-mediated incompatibility (86).

Here, we found the Δ*dinI* and Δ*frlC* mutations elicited ~2-fold increases and the Δ*tatD*, Δ*yhcC*, Δ*yiaL*, and Δ*yrfG* mutations conferred smaller yet statistically significant increases in the SOS response activated by pED208 carriage in the donor cell population. Previous work has shown that DinI is not detected during normal growth of bacterial cells, but is produced at the beginning of the SOS response (37). In the donor population, the production of DinI in response to plasmid carriage-stimulated SOS activation might serve as a negative feedback loop to dampen this chronic stress response in the donor population. While DinI is predicted to act directly through the binding of RecA (38), FrlC and the other Group 1 proteins whose deletions also stimulated the SOS response in the donor population might act indirectly by mitigating other physiological perturbations that coincidentally activate the SOS response. It is enticing to speculate that some members of the F plasmid conjutome evolved as translocatable substrates with the potential to mitigate short-term acquisition costs precisely because of their intrinsic capacity to counteract longer-term fitness costs associated with plasmid carriage.

Interbacterial protein transfer represents one mechanism by which F-like plasmids, and likely other MGEs, can promote their establishment in new transconjugant cells. A second mechanism, also identified in F and a couple of other plasmids, relates to the presence of single-stranded promoters (designated *ssi’s* or F*rpo’s*) within the plasmid MLR regions that can support priming of DNA replication or transcription from ssDNA templates (99–101). *ssi’s* have been shown to be transiently active in new transconjugant cells, resulting in bursts of expression of downstream genes (101, 102). As MLRs are the first regions of plasmids to be conveyed into new transconjugants, *ssi*-directed gene expression may also mitigate plasmid acquisition costs. Thus far, however, only one *ssi* has been identified in the F plasmid MLR, which is located upstream of the *ssb-parB2-psiB-psiA* gene cluster (100). We, therefore, propose that both mechanisms, conjugative protein translocation and transient protein production through *ssi-*mediated transcription, evolved to counteract plasmid acquisition costs, but in different ways. On the one hand, conjugative transfer enables the concomitant transfer of a large repertoire of plasmid and chromosomally encoded proteins whose functions mitigate various physiological stresses accompanying the uptake of the co-transferred and metabolically burdensome MGE. On the other, the expression of *ssi-*regulated genes is temporally delayed by virtue of the requirement to recruit host cell transcription and translation machinery to produce the stress-mitigating proteins. *ssi*-driven expression also supplies only a small subset of effector proteins whose functions are confined mainly to transient SOS suppression.

CRAfT is currently the only assay available for high-throughput screening of protein substrates of conjugation systems, and though robust and exquisitely sensitive, the assay also has intrinsic limitations. In donor cells, Cre-Orf fusions must be stably produced and unfolded prior to translocation through the conjugation channel, and after transfer, the Cre moieties must refold and assemble as a catalytically active tetrameric synaptic complex at *lox* sites (103). Because of these constraints, and the arbitrary cut-offs we used in reiterative rounds of screening to hone the list of chromosomal substrates, we suspect the number of identified protein substrates underrepresents the F system conjutome. Nevertheless, this cohort greatly expands the list of protein substrates reported thus far to be translocated interbacterially through any secretion system (10, 11, 25, 29–32, 34, 35, 104). This cohort now enables further detailed interrogation of the biological consequences of conjugative protein transfer and the TSs conferring protein substrate recognition by conjugation machines.

Toward this latter objective, here we deployed CRAfT to test a prediction that members of the F plasmid conjutome harbor C-terminal TSs, reminiscent of previous findings for relaxases such as *Agrobacterium tumefaciens* VirD2 and RSF1010 MobA that are delivered through the *A. tumefaciens* VirB/VirD4 system (9). Intriguingly, the C termini of F plasmid conjutome substrates parsed into four groups based on overall charge and hydrophobic character, and our analyses of 10 substrates established that TSs can be located internally or C-terminally and function independently of each other or synergistically. We note that the cloned C-termini of substrates with an overall positive charge generally supported the transfer of fused Cre, suggesting that such a motif might constitute a generic TS for the T4SS_ED_. Elsewhere, studies have identified internal motifs constituting TSs among the F-encoded TraI and related relaxases that correspond to structural motifs within the respective helicase domains (25, 26). These findings raise the possibility that the internal TSs carried by F conjutome substrates might require folding of the protein for proper display. Recently, various algorithms have been developed to predict protein substrates of T4SS effector translocator systems (105–108). Once the TSs required for conjugative protein transfer are better understood, this information will be useful to train the algorithms for enhanced predictive power in the identification of the protein repertoires of members of both major T4SS subfamilies.

We previously reported that most or all of the pED208-encoded MLR substrates of the T4SS_ED_ were also translocated through the pKM101 and RP4 conjugation systems (35), and here we expanded this list of promiscuously translocated substrates to include most of the chromosomally encoded members of the F conjutome. Interestingly, we also have found that protein transfer through the T4SS_KM_ is not obligatorily coupled with pKM101 substrate docking and activation of the T4SS_KM_ channel or co-transfer of the DNA substrate into the same recipient cells (35). This may account for the observation that the pKM101 transfer system, but not that of pED208, mediates the transfer of two F plasmid-encoded toxin components of TA systems, SrnB and CcdB (35). Apparently, the pKM101 system but not the F system has the potential to transfer toxins to and kill recipient cells that do not receive the plasmid substrate, potentially facilitating niche establishment by new transconjugant cells. We propose that conjutomes of various conjugation systems consist of a combination of common substrates with functions generally promoting MGE transmission and stress mitigation and other system-specific substrates with specialized functions. If so, TSs conferring conjugative protein transfer may include a combination of generic signals recognizable by many conjugation systems and system-specific signals conferring dedicated transfer. The definition of the conjutomes of other conjugation machines and the nature of TSs conferring conjugative protein transfer remain exciting areas for further exploration.

Finally, the potential for conjugation systems to translocate many proteins suggests new scenarios by which T4SSs evolved their repertoires of specialized effectors. We propose that the ancestral conjugation machines promiscuously translocated many protein substrates, including rolling circle replicases that enabled their evolution as DNA transfer machines. During evolution, conjutomes were honed to include substrates with functions promoting MGE reestablishment and acquisition cost mitigation. With the emergence of eukaryotes, conjugation systems were appropriated as effector translocators to deliver protein effectors into host cells for establishment of pathogenic or symbiotic relationships. Some effector translocators retained while most others lost the capacity to translocate DNA, and effector translocators also potentially retain the capacity to deliver DNA or protein substrates to bacterial targets (109–111). Invariably, the effector translocator systems evolved specialized repertoires of protein substrates through one or more of the following mechanisms: (i) honing of conjutomes to a set of chromosomally encoded proteins whose interkingdom translocation benefited bacterial survival and proliferation in the eukaryotic host, (ii) acquisition of sequences encoding eukaryotic-like proteins or domains that fortuitously carry recognizable TSs, (iii) recombinogenic shuffling between sequences encoding eukaryotic-like proteins or domains and resident conjutome genes with the latter supplying requisite TSs, or (iv) acquired eukaryotic-like proteins or domains ultimately evolved the capacity to bind an adaptor or chaperone capable of physically coupling the protein substrate with a given T4SS. While most extant effector translocators have honed their effector repertoires to fewer than 20 to 30, fascinatingly, the *Legionella* and *Coxiella spp*., Dot/Icm systems traffic several hundreds of effectors, perhaps by retaining functional vestiges of the promiscuous ancestral conjugation system(s) from which these machines arose.

## MATERIALS AND METHODS

### Bacterial strains, plasmids, primers and growth conditions

*E. coli* strains, plasmids, and oligonucleotides used in this study are listed in Table S1. *E. coli* strains were grown in Luria Broth (LB) medium at 30°C for recombineering and 37°C for other applications. Media were supplemented with antibiotics as needed at the final concentrations indicated: spectinomycin (100 µg/mL), streptomycin (100 µg/mL), carbenicillin (100 µg/mL), kanamycin (50 µg/mL), tetracycline (20 µg/mL), gentamycin (10 µg/mL), and chloramphenicol (20 µg/mL).

### Construction of Cre-fused ASKA library

The ASKA library is a collection of plasmids carrying the 3,986 *orfs* constituting the *E. coli* W3110 genome, after the exclusion of pseudogenes and genes for tRNA, rRNA, and IS elements (112). To identify the repertoire of *E. coli* proteins translocated through the pED208-encoded T4SS, we amplified the entire ASKA library of cloned *orfs* using primers complementary to sequences on the vector plasmid pCA24N located immediately upstream and downstream of the cloned *orfs*. We then digested the amplicons with *Sfi*I, which cleaves at sites positioned immediately upstream and downstream of the cloned *orfs* (112). The digested products were electrophoresed through a 1% agarose gel and fragments ranging in size from 100 base pairs (bps) to 2.5 kilobase pairs (kbs) were purified and ligated *en masse* to *Sfi*I-digested pAM38, which carries P_BAD_::*cre* and an *Sfi*I site immediately upstream of *cre’s* stop codon (Fig. 1A). Initial PCR amplification of 12 random *cre-orf* fusions showed that the fused genes exhibited size heterogeneity, and sequence analyses of the amplicons confirmed that *cre* was fused in-frame with cloned *orfs*, thus validating the cloning strategy (Fig. 1A).

### Other plasmid constructions

We assessed the effects of C-terminal residues on the translocation of 10 protein substrates (Can, Fpr, YrfG, RfaH, RspA, SpeE, RhaD, YbeF, EndD, and RspB) by deleting 15–20 C-terminal residues from the Cre-Orf fusions and by fusing Cre to the last 50 residues of these substrates. Plasmids producing Cre fused to the C-terminal truncations and fragments were constructed by inverse PCR using the original *cre-orf* expression plasmids as templates and primers listed in Table S1. The resulting plasmids were verified by sequencing across the entire *cre-orf* fusions. In the original high-throughput screen, the Cre-Orf fusion proteins carried 5 C-terminal residues (GLCGL) resulting from use of the *Sfi*I site for *en masse* cloning (Fig. 1A). To assess its effects on substrate transfer, this motif was deleted from 10 Cre-Orf fusions (Can, Fpr, YrfG, RfaH, RhaD, YbeF, EndD, RspA, RspB, and SpeE) by inverse PCR using primers listed in Table S1.

### Insertion of chromosomal mutations into different host strains by P1 phage transduction

To identify the contributions of translocated proteins to plasmid transfer, P1 phage lysates were prepared of the corresponding mutants from the Keio collection (83). These lysates were used to transfer mutant alleles into MC4100(pED208) by P1 transduction as described previously (113). To determine the effects of chromosomal mutations on the SOS response in pED208-carrying cells, the phage lysates also were used to introduce mutant alleles into the SOS reporter MG1655 Δ*attλ*::P*_sulA_mCherry-*FRT*cat*FRT harboring pED208 (34).

### High-throughput CRAfT screen

We patched 8,150 colonies obtained by transformation of the *cre-orf* plasmid library into MC4100(pED208) onto LB plates supplemented with carbenicillin and spectinomycin. Plates were incubated overnight at 37°C, and patched strains were successively inoculated into 96-well plates containing 75 µL of LB media. Strains were grown in the presence of arabinose (0.2% final concentration) to induce expression of the *cre-orf* gene fusions for 2 h at 37°C. The recipient strain CSH26Cm:: LTL, which contains a *loxP-tet^R^-loxP* cassette interrupting a *chl^R^* gene on the bacterial chromosome (114), was prepared by growth overnight in liquid LB media with tetracycline, dilution 1:50 in fresh LB broth, and incubation with shaking for 1.5 h (OD_600_ ~0.3) at 37°C. Aliquots (75 µL) of the recipient cell culture were dispensed into microtiter plate wells containing the MC4100(pED208) strains induced for *cre-orf* expression (donor strains). Mating mixes were incubated at 37°C for 20 h, and then 5 µL aliquots were dispensed onto chloramphenicol-containing media selective for *loxP* recombinants. Plates were incubated at 37°C for 24 h, and colonies were patched onto LB plus tetracycline to confirm *loxP* excision and LB plus spectinomycin to assay for co-transfer of pED208.

### Quantitative CRAfT

Quantitative CRAfT was carried out as previously described (35). Briefly, donor and recipient strains were grown overnight in LB broth with antibiotic selection at 37 ◦C with shaking. Overnight cultures were subcultured 1:50 into fresh LB broth (2 mL) at 37 ◦C with shaking for 1.5 h (OD_600_ ~0.3). Donor and recipient cells (10 µL each) were mixed, spotted onto sterile nitrocellulose filters on LB plates containing 0.2% arabinose (final volume) for induction of *cre* gene fusions from the P_BAD_ promoter, and plates were incubated at 37°C for 20 h. Cells were resuspended from the filter in LB broth and serially diluted, and donors, recipients, and *loxP* recombinants were selected on LB agar plates containing the appropriate antibiotics. The frequency of Cre recombination was calculated as the number of recombinants (Rcs) per donor (D). Experiments were performed at least three times in triplicate, and results are reported as the mean frequency of transfer with standard error or mean (SEM).

### Conjugative DNA transfer assay

Donor and recipient cells were grown overnight at 37°C in the presence of the appropriate antibiotics, diluted 1:50 in fresh antibiotic-free LB media, and incubated without shaking for 1.5 h. 75 µL of donor and recipient cell cultures were mixed and incubated without shaking for 1.5 h. Mating mixtures were serially diluted and plated onto LB agar containing antibiotics selective for transconjugants (Tcs) and donors. The frequency of DNA transfer was calculated by dividing the number of transconjugant colonies by the number of donor colonies (Tcs/D). Mating experiments were performed at least three times in triplicate, and results are reported as the mean frequency of transfer with standard error of the mean (SEM).

### Properties of C-terminal regions of chromosomal substrates

Physical properties of the C-terminal 50 residues of the 32 chromosomal substrates of the pED208-encoded T4SS were assessed using the algorithm https://www.bachem.com/knowledge-center/peptide-calculator/. Substrates were assigned into one of four groups based on overall charge and hydrophobicity of the C-terminal regions.

### Detection of the mating-induced SOS response by flow-cytometry

Induction of the SOS response during mating was quantitated at the single-cell level by flow cytometry as previously described (34, 85). An *E. coli* strain expressing red-fluorescent protein mCherry from the SOS-inducible *sulA* promoter (86) (Δ*attλ*::P*_sulA_*-mCherry*)* served as a recipient during mating (86). Matings between pED208-carrying donor strains and the SOS reporter strain were carried out as described above, and mating mixtures were harvested by centrifugation and resuspended in 1 mL filter sterilized M9 minimal salts medium. Mating mixtures were diluted 1:25 to 1:50, samples were subjected to flow cytometry using an LSR Fortessa flow cytometer (BD Biosciences), and data were analyzed with BD FACSDivaTM and FlowJo software. A “red” gate was set using the *lexA3* mutant (SOS-induction deficient); cells to the right of the gate are considered SOS induced (34, 85). For these analyses, 10^6^ events were collected per strain, with each strain assayed three times in three independent experiments. The percentage of SOS-induced cells was calculated by dividing the number of SOS-induced cells with 10^6^ events multiplied by 100 [(# of SOS-induced cells/10^6^) × 100]. The relative SOS induction was determined by dividing the percentage of SOS induction for matings between MC4100(pED208) strains and the SOS reporter strain by the percentage of SOS induction of the control mix (plasmid-free MC4100 × SOS reporter). For redundant plasmid transfer, the relative induction of SOS was determined by dividing the percent of SOS induction of the MC4100(pED208) strain carrying Δ*attλ*::P*_sulA_*-mCherry by that of the plasmid-free MC4100 SOS reporter (34).

## SUPPLEMENTAL MATERIAL

Supplemental material msphere.00354-24-s0001.pdf Supplemental tables and figures.

ASM does not own the copyrights to Supplemental Material that may be linked to, or accessed through, an article. The authors have granted ASM a non-exclusive, world-wide license to publish the Supplemental Material files. Please contact the corresponding author directly for reuse.
