# Supplementary material for: The F plasmid conjutome: the repertoire of E. coli proteins translocated through an F-encoded type IV secretion system
Source: mSphere. 2024 Jun 28;9(7):e00354-24. doi: 10.1128/msphere.00354-24 (PMC11288057; doi:10.1128/msphere.00354-24)
Supplement: Supplemental material — Supplemental tables and figures. [file msphere.00354-24-s0001.pdf]

Table S1. Strains, plasmids, and primers used in this study.

Strains	Description	Source (references)
MC4100	*araD139* λ^- *e14*$^-$ *Δ(argF-lac)169 rpsL150*(strR) *flhD5301 Δ(fimB-fimE)632* F$^-$	Lab stock
JCM158	MC4100 *ara*$^{R/-}$; spontaneous arabinose resistant	(1)
CSH26Cm::LTL	CSH26 *galK*::*cat*::*loxP*-*tet*^{R}-*loxP*	(2)
DH5α	*endA1 glnV44 thi-1* *recA1 relA1 gyrA96 deoR nupG purB20* φ80d*lacZ*ΔM15 Δ(*lacZYA-argF*)U169, hsdR17($r_K^-m_K^+$), λ$^-$ F$^-$	Lab stock
MG1655	*rph-1* λ F$^-$	Lab stock
SMR17962	MG1655 Δ*attλ*::P$_{sulA}$*mCherry*- FRT*cat*FRT	(3)
SMR17966	MG1655 Δ*attλ*::P$_{sulA}$*mCherry*- FRT *lexA3*(Ind-) *malB*::Tn*9::cat*	(3)
AA69	MC4100 pED208::*spc*^{R}	(4)
AA1165	MG1655 Δ*attλ*::P$_{sulA}$*mCherry*- FRT*cat*FRT pED208::*spc*^{R}	(4)
E. coli S17-1	RP4-2-Tc::Mu Km::Tn*7 recA* Tm^R Sm^R	(5)
JW1048	Δ*dinI*::FRT*kan*FRT	(6)
JW5669	Δ*frlC*::FRT*kan*FRT	(6)
JW2654	Δ*nrdE*::FRT*kan*FRT	(6)
JW2245	Δ*nudI*::FRT*kan*FRT	(6)
JW2688	Δ*recX*::FRT*kan*FRT	(6)
JW5931	Δ*tatD*::FRT*kan*FRT	(6)
JW3178	Δ*yhcC*::FRT*kan*FRT	(6)
JW3548	Δ*yiaL*::FRT*kan*FRT	(6)
JW5265	Δ*yrfG*::FRT*kan*FRT	(6)
AA2246	MC4100 Δ*dinI*::FRT*kan*FRT pED208::*spc*^{R}	AA69 x P1 (JW1048)
AA2247	MC4100 Δ*frlC*::FRT*kan*FRT pED208::*spc*^{R}	AA69 x P1 (JW5669)
AA2327	MC4100 Δ*nrdE*::FRT*kan*FRT pED208::*spc*^{R}	AA69 x P1 (JW2654)
AA2328	MC4100 Δ*nudI*::FRT*kan*FRT pED208::*spc*^{R}	AA69 x P1 (JW2245)
AA2245	MC4100 Δ*recX*::FRT*kan*nFRT pED208::*spc*^{R}	AA69 x P1 (JW2688)
AA2263	MC4100 Δ*tatD*::FRT*kan*FRT pED208::*spc*^{R}	AA69 x P1 (JW5931)
AA2329	MC4100 Δ*yhcC*::FRT*kan*FRT pED208::*spc*^{R}	AA69 x P1 (JW3178)
AA2249	MC4100 Δ*yiaL*::FRT*kan*FRT pED208::*spc*^{R}	AA69 x P1 (JW3548)
AA2330	MC4100 Δ*yrfG*::FRT*kan*FRT pED208::*spc*^{R}	AA69 x P1 (JW5265)
AA2251	MG1655 Δ*attλ*::P$_{sulA}$*mCherry*-FRT*cat*FRT Δ*dinI*:: FRT*kan*FRT pED208::*spc*^{R}	AA1165 x P1 (JW1048)
AA2252	MG1655 Δ*attλ*::P$_{sulA}$*mCherry*-FRT*cat*FRT Δ*frlC*:: FRT*kan*FRT pED208::*spc*^{R}	AA1165 x P1 (JW5669)
AA2317	MG1655 Δ*attλ*::P$_{sulA}$*mCherry*-FRT*cat*FRT Δ*nrdE*:: FRT*kan*FRT pED208::*spc*^{R}	AA1165 x P1 (JW2654)
AA2325	MG1655 Δ*attλ*::P$_{sulA}$*mCherry*-FRT*cat*FRT Δ*nudI*:: FRT*kan*FRT pED208::*spc*^{R}	AA1165 x P1 (JW2245)

AA2250	MG1655 Δ*attλ*::P_{sulA}*mCherry*-FRT*cat*FRT Δ*recX*:: FRT*kan*FRT pED208::*spc*^{R}	AA1165 x P1 (JW2688)
AA2253	MG1655 Δ*attλ*::P_{sulA}*mCherry*-FRT*cat*FRT Δ*tatD*:: FRT*kan*FRT pED208::*spc*^{R}	AA1165 x P1 (JW5931)
AA2319	MG1655 Δ*attλ*::P_{sulA}*mCherry*-FRT*cat*FRT Δ*yhcC*:: FRT*kan*FRT pED208::*spc*^{R}	AA1165 x P1 (JW3178)
AA2254	MG1655 Δ*attλ*::P_{sulA}*mCherry*-FRT*cat*FRT Δ*yiaL*:: FRT*kan*FRT pED208::*spc*^{R}	AA1165 x P1 (JW3548)
AA2326	MG1655 Δ*attλ*::P_{sulA}*mCherry*-FRT*cat*FRT Δ*yrfG*:: FRT*kan*FRT pED208::*spc*^{R}	AA1165 x P1 (JW5265)
**Plasmids**		
pED208	A natural derivative of F_0-*lac*$^+$ IncFV	(7)
pED208::*spc*^{R}	Spc^R, pED208 bearing *spc*^{R}	(8)
pED208:: *spc*^{R}Δ*traD*	Spc^R, pED208::*spc*^{R} deleted of *traD*	(8)
pED208::*spc*^{R}Δ*traI*	Spc^R, pED208::*spc*^{R} deleted of *traI*	(4)
pBAD24	Crb^R; ColE1 plasmid with arabinose-inducible P_{BAD} promoter	(9)
pAM38 (pcre)	Crb^R; pBAD24 with P_{BAD}::*cre*	(4)
pAM37	Crb^R; pBAD24 with P_{BAD}::*cre-psiB*	(4)
pAM39	Crb^R; pBAD24 with P_{BAD}::*cre-traI*	(4)
pBD-cre-dinI	Crb^R; pBAD24 with P_{BAD}::*cre-dinI*	This study
pBD-cre-frlC	Crb^R; pBAD24 with P_{BAD}::*cre-frlC*	This study
pBD-cre-nrdE	Crb^R; pBAD24 with P_{BAD}::*cre-nrdE*	This study
pBD-cre-nudI	Crb^R; pBAD24 with P_{BAD}::*cre-nudI*	This study
pBD-cre-tatD	Crb^R; pBAD24 with P_{BAD}::*cre-tatD*	This study
pBD-cre-yhcC	Crb^R; pBAD24 with P_{BAD}::*cre-yhcC*	This study
pBD-cre-yiaL	Crb^R; pBAD24 with P_{BAD}::*cre-yiaL*	This study
pBD-cre-yrfG	Crb^R; pBAD24 with P_{BAD}::*cre-yrfG*	This study
pBD-cre-can	Crb^R; pBAD24 with P_{BAD}::*cre-can*	This study
pBD-cre-entD	Crb^R; pBAD24 with P_{BAD}::*cre-entD*	This study
pBD-cre-fpr	Crb^R; pBAD24 with P_{BAD}::*cre-fpr*	This study
pBD-cre-gadA	Crb^R; pBAD24 with P_{BAD}::*cre-gadA*	This study

pBD-cre-gpmM	Crb^R; pBAD24 with P_{BAD}::*cre-gpmM*	This study
pBD-cre-hemG	Crb^R; pBAD24 with P_{BAD}::*cre-hemG*	This study
pBD-cre-rspA	Crb^R; pBAD24 with P_{BAD}::*cre-rspA*	This study
pBD-cre-sodA	Crb^R; pBAD24 with P_{BAD}::*cre-sodA*	This study
pBD-cre-speE	Crb^R; pBAD24 with P_{BAD}::*cre-speE*	This study
pBD-cre-yciE	Crb^R; pBAD24 with P_{BAD}::*cre-yciE*	This study
pBD-cre-yihT	Crb^R; pBAD24 with P_{BAD}::*cre-yihT*	This study
pBD-cre-yihV	Crb^R; pBAD24 with P_{BAD}::*cre-yihV*	This study
pBD-cre-yphB	Crb^R; pBAD24 with P_{BAD}::*cre-yphB*	This study
pBD-cre-rfaH	Crb^R; pBAD24 with P_{BAD}::*cre-rfaH*	This study
pBD-cre-ybeF	Crb^R; pBAD24 with P_{BAD}::*cre-ybeF*	This study
pBD-cre-cbeA	Crb^R; pBAD24 with P_{BAD}::*cre-cbeA*	This study
pBD-cre-miaB	Crb^R; pBAD24 with P_{BAD}::*cre-miaB*	This study
pBD-cre-rhaD	Crb^R; pBAD24 with P_{BAD}::*cre-rhaD*	This study
pBD-cre-tabA	Crb^R; pBAD24 with P_{BAD}::*cre-tabA*	This study
pBD-cre-yagB	Crb^R; pBAD24 with P_{BAD}::*cre-yagB*	This study
pBD-cre-yfjZ	Crb^R; pBAD24 with P_{BAD}::*cre-yfjZ*	This study
pBD-cre-rspB	Crb^R; pBAD24 with P_{BAD}::*cre-rspB*	This study
pBD-cre-ydhF	Crb^R; pBAD24 with P_{BAD}::*cre-ydhF*	This study
pBD-cre-yfjX	Crb^R; pBAD24 with P_{BAD}::*cre-yfjX*	This study
pKM101::*spc*	pKM101 derivative	(10)
pYGL160	pBAD24 with P_{BAD}::$traK_{HA}$-$traJ_{Strep}$-cre-$traI_{His}$ Crb^R	(11)
pYGL258	pSC101 with P_{BAD}::*cre-mob*A_{His}	(11)
pcre-can_{N197}	pBAD24 with P_{BAD}::*cre-can*$_{N197}$ (encodes N-terminal 197 residues of Can)	This study
pcre-fpr_{N225}	pBAD24 with P_{BAD}::*cre-fpr*$_{N225}$	This study
pcre-$yrfG_{N202}$	pBAD24 with P_{BAD}::*cre-yrfG*$_{N202}$	This study
pcre-$rfaH_{N147}$	pBAD24 with P_{BAD}::*cre-rfaH*$_{N147}$	This study

pcre-speE$_{N269}$	pBAD24 with P_{BAD}::*cre-speE*$_{N269}$	This study
pcre-rspA$_{N379}$	pBAD24 with P_{BAD}::*cre-rspA*$_{N379}$	This study
pcre-rhaDN$_{249}$	pBAD24 with P_{BAD}::*cre-rhaD*$_{N249}$	This study
pcre-ybeF$_{N292}$	pBAD24 with P_{BAD}::*cre-ybeF*$_{N292}$	This study
pcre-entD$_{N181}$	pBAD24 with P_{BAD}::*cre-entD*$_{N181}$	This study
pcre-rspB$_{N1314}$	pBAD24 with P_{BAD}::*cre-rspB*$_{N314}$	This study
pcre-can$_{C50}$	pBAD24 with P_{BAD}::*cre-can*$_{C50}$ (Encodes Cre fused to C-terminal 50 residues of Can)	This study
pcre-fpr$_{C50}$	pBAD24 with P_{BAD}::*cre-fpr*$_{C50}$	This study
pcre-yrfG$_{C50}$	pBAD24 with P_{BAD}::*cre-yrfG*$_{C50}$	This study
pcre-rfaH$_{C50}$	pBAD24 with P_{BAD}::*cre-rfaH*$_{C50}$	This study
pcre-speE$_{C50}$	pBAD24 with P_{BAD}::*cre-speE*$_{C50}$	This study
pcre-rspA$_{C50}$	pBAD24 with P_{BAD}::*cre-rspA*$_{C50}$	This study
pcre-rhaD$_{C50}$	pBAD24 with P_{BAD}::*cre-rhaD*$_{C50}$	This study
pcre-ybeF$_{C50}$	pBAD24 with P_{BAD}::*cre-ybeF*$_{C50}$	This study
pcre-entD$_{C50}$	pBAD24 with P_{BAD}::*cre-entD*$_{C50}$	This study
pcre-RspB$_{C50}$	pBAD24 with P_{BAD}::*cre-rspB*$_{C50}$	This study
pYGL690	pBAD24 with P_{BAD}::*cre-fprΔGLCGL* (Encodes Cre-Fpr deleted of C-terminal GLCGL motif)	This study
pYGL691	pBAD24 with P_{BAD}::*cre-rspBΔGLCGL*	This study
pYGL692	pBAD24 with P_{BAD}::*cre-entDΔGLCGL*	This study
pYGL693	pBAD24 with P_{BAD}::*cre-yrfGΔGLCGL*	This study
pYGL694	pBAD24 with P_{BAD}::*cre-ybeFΔGLCGL*	This study
pYGL695	pBAD24 with P_{BAD}::*cre-canΔGLCGL*	This study
pYGL696	pBAD24 with P_{BAD}::*cre-rfaHΔGLCGL*	This study
pYGL697	pBAD24 with P_{BAD}::*cre-rhaDΔGLCGL*	This study
pYGL698	pBAD24 with P_{BAD}::*cre-speEΔGLCGL*	This study
pYGL699	pBAD24 with P_{BAD}::*cre-rspAΔGLCGL*	This study

Primers	Sequence (5' to 3')[a]	Purpose
P1_lib_F	tacggatccggccctgagGG	ASKA library DNA fragments
P2_lib-*Sfi*I_R	cctttactgcGGCCGCATAGGCC	ASKA library DNA fragments
P3_*cre*-*Sfi*I_F	aaccta GGCCTATGCGGCCtg taatcctctagagtcgacctgcaggc	Amplify p*cre* to insert ASKA library DNA fragments
P4_*cre*-*Sfi*I_R	taacacGGCCCTCAGGGCCgcatcgccatct tccagcagg	Amplify p*cre* to insert ASKA library DNA fragments
P5_BAD24_F	caaagcgggaccaaagccatgac	Check inserts in p*cre*
P6_BAD24_R	cagttccctactctcgcatgggg	Check inserts in p*cre*
AA39_BAD24sq_F	aagtccacattgattatttgcacg	Sequence inserts in p*cre-orf* plasmids
AA40_BAD24sq_R	cacttctgagttcggcatgg	Sequence inserts in p*cre-orf* plasmids
AA136_cresq_F	gtcgagcgatggatttccg	Sequence inserts in p*cre-orf* plasmids
AA1196_TSdel*Xba*I_F	taatccTCTAGAgtcgacctgcag	Deletions of last 15 to 25 codons from *can*, *entD*, *fpr*, *rfaH*, *rhaD*, *rspA*, *rspB*, *speE*, *ybeF* and *yrfG* in cre-orf plasmids
AA1197_*can*TSdel*Xba*I_R	atacatTCTAGAttagttggtggcggtaacatccag	Deletion of last 23 codons from *cre-can*
AA1218_*entD*TSdl*Xba*I_R	atacatTCTAGAttaaatgatgacctgctgtttattcc ag	Deletion of last 25 codons from *cre-entD*
AA1198_*fpr*TSdel*Xba*I_R	atacatTCTAGAttacagcaactgttgtgtatcgcgc	Deletion of last 23 codons from *cre-fpr*
AA1200_*rfaH*TSdel*Xba*I_ R	atacatTCTAGAttaattaataagattaagcaatagc atggagcgagc	Deletion of last 15 codons from *cre-rfaH*
AA1217_*rhaD*TSdl*Xba*I_R	atacatTCTAGAttactgtttcatgccgcccatc	Deletion of last 25 codons from *cre-rhaD*
AA1214_*rspA*TSdl*Xba*I_R	atacatTCTAGAttacttttcatcgaattcgataccaa gacc	Deletion of last 25 codons from *cre-rspA*
AA1215_*rspB*TSdl*Xba*I_R	atacatTCTAGAttactggaaatcaaacgtatgggt	Deletion of last 25 codons from *cre-rspB*
AA1201_*speE*TSdel*Xba*I_ R	atacatTCTAGAttaatggattgccggattgtaataa cgg	Deletion of last 19 codons from *cre-speE*
AA1216_*ybeF*TSdl*Xba*I_R	atacatTCTAGAttagaaatctatttgctcctcatttaa ggacg	Deletion of last 25 codons from *cre-ybeF*
AA1199_*yrfG*TSdel*Xba*I_ R	atacatTCTAGAttactcggcaatcccggaatcag	Deletion of last 20 codons from *cre-yrfG*
AA1202_N-termdel*Kpn*I_R	atactaGGTACCatcgccatcttccagcaggc	*cre* fused to last 50 codons of *can*, *entD*, *fpr*, *rfaH*, *rhaD*, *rspA*, *rspB*, *speE*, *ybeF* and *yrfG*
AA1203_*can*Ndel*Kpn*I_F	agaagaGGTACCgggcagaaagttaccattcagg	*cre* fused to last 50 codons of *can*
AA1223_*entD*Ndl*Kpn*I_F	agaagaGGTACCgcaagtgagatccaaactgatg	*cre* fused to last 50 codons of *entD*

AA1204_*fpr*Ndl*Kpn*I_F	agaagaGGTACCggcctgccgatgaataaagaaacc	*cre* fused to last 50 codons of *fpr*
AA1206_*rfaH*Ndl*Kpn*I_F	agaagaGGTACCggtgataaggtgattatccgaaggc	*cre* fused to last 50 codons of *rfaH*
AA1222_*rhaD*Ndl*Kpn*I_F	agaagaGGTACCttcggtttaatcgacaccgcg	*cre* fused to last 50 codons of *rhaD*
AA1219_*rspA*Ndl*Kpn*I_F	agaagaGGTACCcacaactggactttcgataacgg	*cre* fused to last 50 codons of *rspA*
AA1220_*rspB*Ndl*Kpn*I_F	agaagaGGTACCccgatcgttatcgactggtta	*cre* fused to last 50 codons of *rspB*
AA1207_*speE* Ndl*Kpn*I _F	agaagaGGTACCaacgacgccttacgccatctc	*cre* fused to last 50 codons of *speE*
AA122_*ybeF*Ndl*Kpn*I_F	AGAAGA ggtacc tat aac ctg ttt agc cgc tgc	*cre* fused to last 50 codons of *ybeF*
AA1205_*yrfG*Ndl*Kpn*I_F	agaagaGGTACCgatgacagcgaagcgattctcg	*cre* fused to last 50 codons of *yrfG*
dGLCGL_F	taatcctctagagtcgacctgcaggcatg	*cre-orf* fusions deleted of codons for GLGCL
Fpr_R	ttaccagtaatgctccgctgtcatatggcc	*cre-fpr* lacking GLGCL codons
Can_R	ttatttgtggttggcgtgtttcagcttgaggttg	*cre-can* lacking GLGCL codons
RfaH_R	ttagagtttgcggaactcggtattcttcacactg	*cre-rfaH* lacking GLGCL codons
SpeE_R	ttaggacggctgtgaagccagtgcgtc	*cre-speE* lacking GLGCL codons
RspB_R	ttattcagaaaaagtgagtaagactttgcagcaatgtttttgatc	*cre-rspB* lacking GLGCL codons
RhaD_R	ttacagcgccagcgcactggcgag	*cre-rhaD* lacking GLGCL codons
EntD_R	ttaatcgtgttggcacagcgttatgactatcttttc	*cre-entD* lacking GLGCL codons
YrfG_R	ttacattagcgaggggatcaggcggcg	*cre-yrfG* lacking GLGCL codons
YbeF_R	ttaaaatgcattacggataacatctattactccgtgcag	*cre-ybeF* lacking GLGCL codons
Cre-rspA-dGLCGL-I_F	cataagattagcggatcctacctgacgc	*cre-rspA* lacking GLGCL codons
Cre-rspA-dGLCGL-I_R	tcttctctcatccgccaaaacagccttaccagttccacagcgtgccatcttc	*cre-rspA* lacking GLGCL codons
Cre-rspA-dGLCGL-V_F	ggctgttttggcggatgagagaagattttc	*cre-rspA* lacking GLGCL codons
Cre-rspA-dGLCGL-V_R	tcaggtaggatccgctaatcttatggataaaaatg	*cre-rspA* lacking GLGCL codons

[a]Restriction sites used for cloning are in uppercase.

References

1. Wu T, Malinverni J, Ruiz N, Kim S, Silhavy TJ, Kahne D. 2005. Identification of a multicomponent complex required for outer membrane biogenesis in *Escherichia coli*. Cell 121:235-45.
2. Lang S, Gruber K, Mihajlovic S, Arnold R, Gruber CJ, Steinlechner S, Jehl MA, Rattei T, Frohlich KU, Zechner EL. 2010. Molecular recognition determinants for type IV secretion of diverse families of conjugative relaxases. Mol Microbiol 78:1539-55.

3. Nehring RB, Gu F, Lin HY, Gibson JL, Blythe MJ, Wilson R, Bravo Nunez MA, Hastings PJ, Louis EJ, Frisch RL, Hu JC, Rosenberg SM. 2016. An ultra-dense library resource for rapid deconvolution of mutations that cause phenotypes in *Escherichia coli*. Nucleic Acids Res 44:e41.
4. Al Mamun AAM, Kishida K, Christie PJ. 2021. Protein transfer through an F plasmid-encoded type IV secretion system suppresses the mating-induced SOS response. mBio 12:e0162921.
5. Simon R, Priefer U, Puhler A. 1983. A broad host range mobilization system for *in vivo* genetic engineering: transposon mutagenesis in Gram negative bacteria. Bio/Technology 1:37-45.
6. Baba T, Ara T, Hasegawa M, Takai Y, Okumura Y, Baba M, Datsenko KA, Tomita M, Wanner BL, Mori H. 2006. Construction of *Escherichia coli* K-12 in-frame, single-gene knockout mutants: the Keio collection. Mol Syst Biol 2:2006 0008.
7. Falkow S, Baron LS. 1962. Episomic element in a strain of *Salmonella Typhosa*. J Bacteriol 84:581-9.
8. Hu B, Khara P, Song L, Lin AS, Frick-Cheng AE, Harvey ML, Cover TL, Christie PJ. 2019. *In situ* molecular architecture of the *Helicobacter pylori* Cag Type IV Secretion System. mBio 10(3):e00849-19.
9. Guzman LM, Belin D, Carson MJ, Beckwith J. 1995. Tight regulation, modulation, and high-level expression by vectors containing the arabinose P_{BAD} promoter. J Bacteriol 177:4121-30.
10. Gordon JE, Costa TRD, Patel RS, Gonzalez-Rivera C, Sarkar MK, Orlova EV, Waksman G, Christie PJ. 2017. Use of chimeric type IV secretion systems to define contributions of outer membrane subassemblies for contact-dependent translocation. Mol Microbiol 105:273-293.
11. Li YG, Christie PJ. 2020. The TraK accessory factor activates substrate transfer through the pKM101 type IV secretion system independently of its role in relaxosome assembly. Mol Microbiol 114:214-229.

Table S2. **Properties of C-terminal 25 residues**.

Protein	C-terminal 25 residues	Isoelectric point	Pos. charged residues	Net charge pH 7	Average hydrophilicity
1. Positively charged (net charge ≥1 and hydrophilicity ≥-0.1)					
YciE	LIQNIPQTTEKFLIRSETDGVEAKK	9.68	4	1.00	0.41
FrlC	YMNEPRLYARQALERFRALLPEDER	9.53	5	1.00	0.53
SodA	RPDYIKEFWNVVNWDEAAARFAAKK	10.08	5	2.00	0.24
YdhF	EAETLKMTRQQWFRIRKAALGYDVP	10.76	5	3.00	0.23
YihT	MLRDVCAPKLQQLGDIVDEMMAKRR	9.89	5	1.96	0.45
YihV	KCTRPGGRAGIPDCDQTRSFLSLFV	10.14	4	2.91	0.14
**YrfG**[a]	SGIAEKQYQRHPSLNDYRRLIPSLM	10.67	5	3.09	0.14
NudI	WVKPEDLVHYDLNVATRKTLRLKGL	10.51	5	3.09	0.13
YiaL	RPGCIMQTASEIRKIVVKVALTALN	11.53	4	3.95	-0.10
TabA	EVHKPLCAVGAPAQVRKAVVKMLMA	10.88	5	4.05	-0.10
GadA	KASLKYLSDHPKLQGIAQQNSFKHT	10.70	6	4.18	0.09
**RfaH**[a]	RSMLLLNLINKEIKHSVKNTEFRKL	11.73	7	5.09	0.26
**Fpr**[a]	LLKETRQMTKHLRRRPGHMTAEHYW	12.04	9	5.27	0.29
**Can**[a]	TNRETLEQRYRHGISNLKLKHANHK	11.56	9	5.27	0.51
2. Hydrophobic (hydrophilicity <-0.1 and net charge <1)					
CbeA	TCKADTLSSCDYVYLAVYPTPEMKN	6.19	2	-0.09	-0.13
NrdE	TLYYIRLRQMALEGTEIEGCVSCAL	6.33	2	-0.09	-0.26
GpmM	APTMLSLMGMEIPQEMTGKPLFIVE	4.63	1	-1.00	-0.28
TatD	WRGEDAAWLAATTDANVKTLFGIAF	7.04	2	0.00	-0.31
**RspA**[a]	LAAKYPYEPAYLPVARLEDGTLWNW	6.99	2	-0.00	-0.38
YfjZ	NGFTCEADTLGSCGYVVYIAVYPTQR	6.14	1	-0.09	-0.42
**SpeE**[a]	YNPAIHTAAFALPQYLQDALASQPS	7.77	1	0.09	-0.55
3. Positively charged and hydrophobic (net charge ≥1 and hydrophilicity <-0.1)					
**YbeF**[a]	SIHYNKFSLRDPILHGVIDVIRNAF	10.49	5	2.18	-0.26
**RhaD**[a]	TISREELIALGKRFGVTPLASALAL	11.34	3	2.00	-0.15
YagB	TLGSHGYVVYIAILNRHGFNRHLRVI	12.18	6	4.27	-0.54
4. No predicted signals (net charge <1 and hydrophilicity >-0.1)					
DinI	KEDKQRISEILQETWESADDWFVSE	4.13	3	-3.99	0.69
YphB	EGGDLIALAPGESTTSEMSLRVEWL	3.99	1	-2.99	0.08
YhcC	ELDRYLNEHGVQGSALGRPWLPPTE	5.49	3	-0.91	0.12

**RspB**[a]	HVADAISLFEQDQKHCCKVLLTFSE	6.04	4	-0.91	-0.02
MiaB	VAETPESVIARTRKENDLGVGYYQP	7.05	3	0.00	0.29
HemG	VYTDWEQVANFAREIAHLTDKPTL	7.79	4	0.09	0.06
YfjX	TEHYYRLRDYALQHPESSAIMRIID	7.88	5	0.18	0.09
**EntD**[a]	HRENEMFAVHWQIKEKIVITLCQHD	7.42	6	0.23	0.03

[a]Proteins in bold were analyzed for the presence of C-terminal or internal translocation signals.

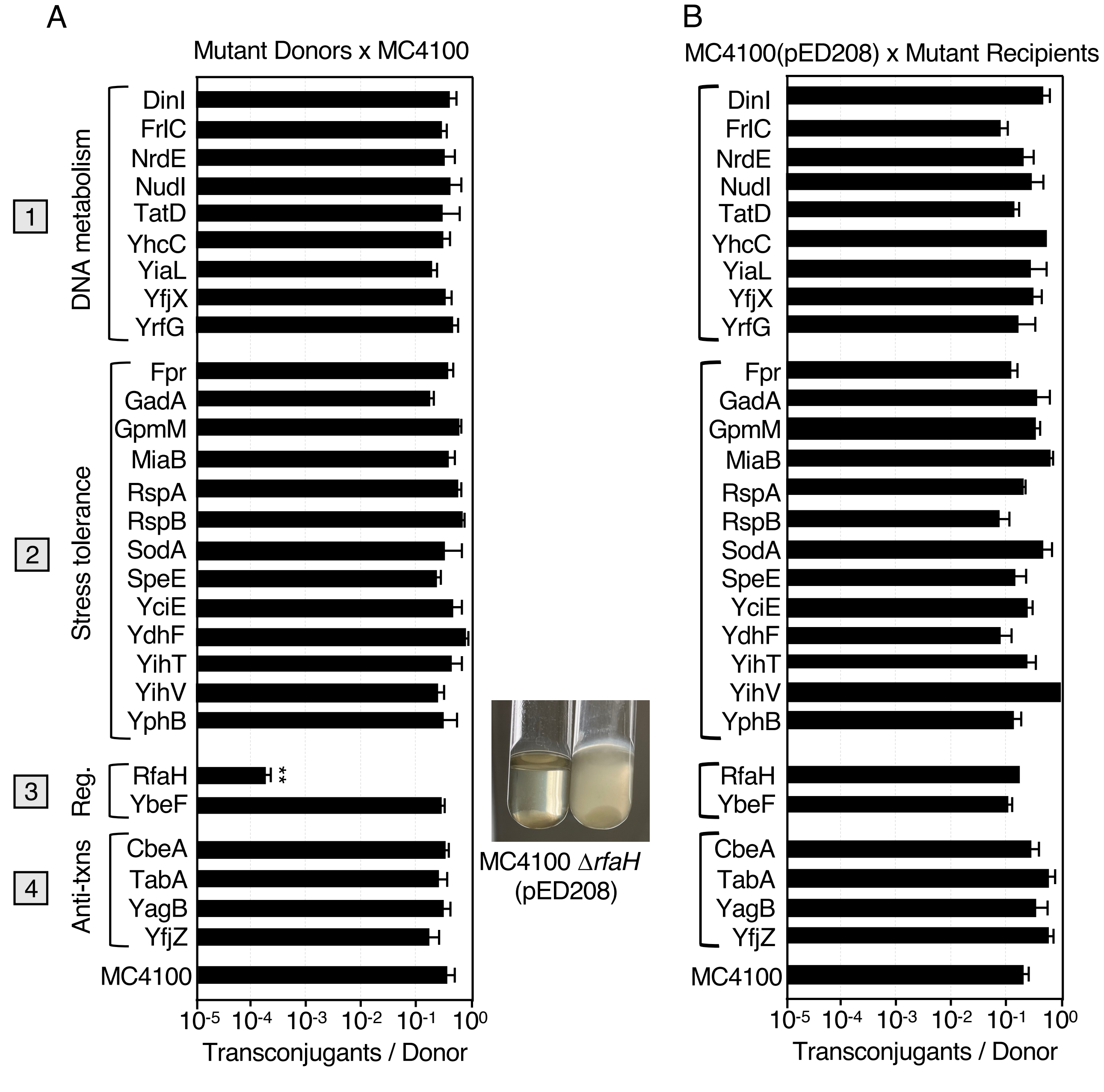

Fig. S1. Donor proficiency of MC4100 variants serving as donors or recipients. Left: pED208-carrying donors deleted of the genes for the proteins listed were mated with MC4100-Rif^R recipients. Right: MC4100(pED208) donors were mated with recipients deleted of the genes for the proteins listed. Strains bearing the mutations were from the Keio collection. Matings were carried out in liquid culture for 90 min and pED208 transfer frequencies are reported as Transconjugants per Donor. Experiments were performed three times in triplicate, with the mean of the three experiments represented by bar lengths and standard deviations by error bars. *P* value for Δ*rfaH* mutant donor transfer frequency denotes comparison with WT MC4100 transfer frequency: **, $P < 0.005$. Tube cultures: MC4100 or the Δ*rfaH* mutant harboring pED208 were grown overnight without shaking and visualized for ED208 pilus-mediated aggregation as a proxy for pilus production.

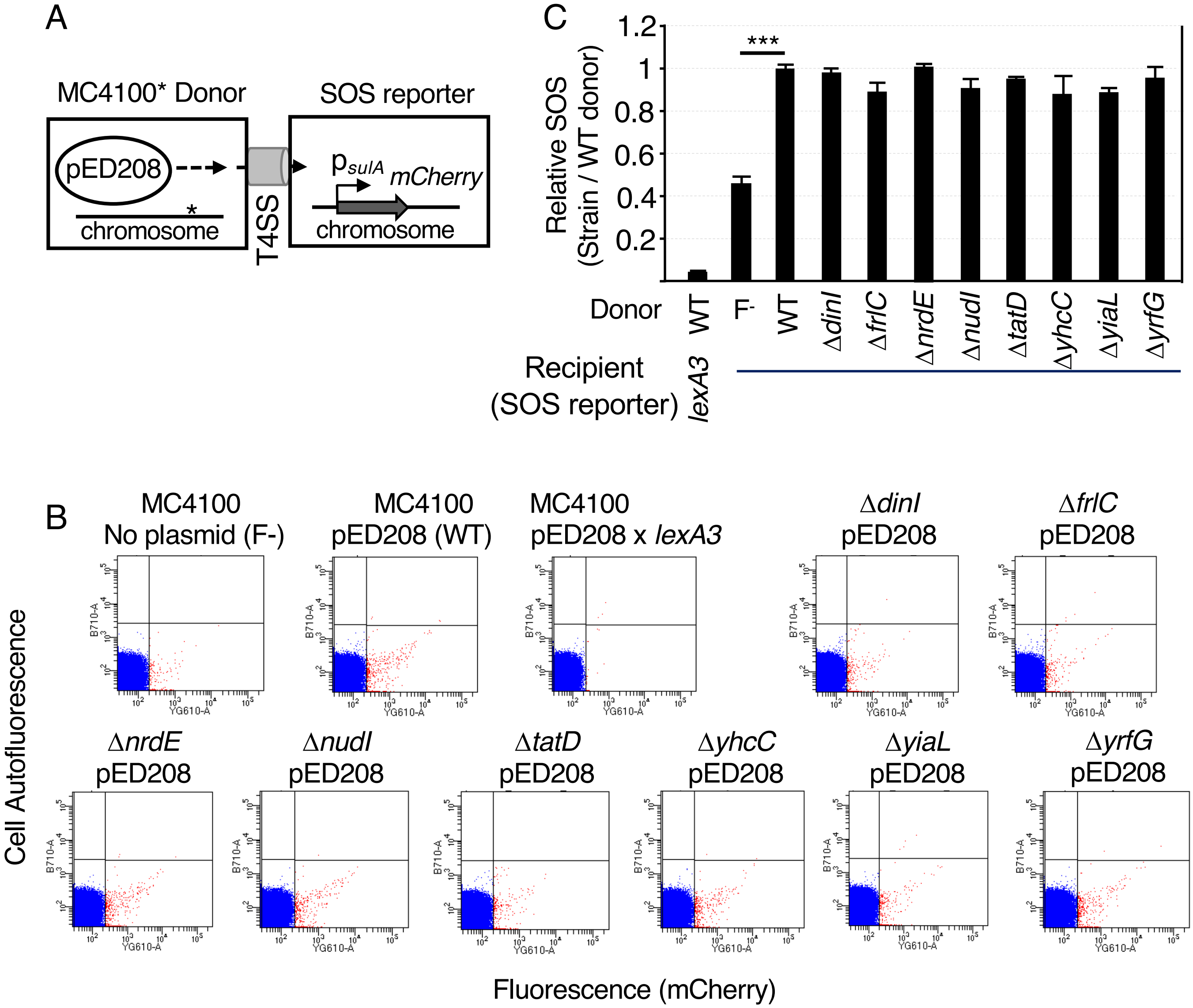

Fig. S2. Donors harboring deletions of DNA metabolism genes do not elicit the SOS response in matings with the SOS reporter strain. A. Schematic of MC4100 mutant strains carrying IncFV pED208 mated with the SOS reporter. Asterisk denotes deletions of the Group I genes with known or predicted functions in DNA metabolism. **B.** Representative examples of flow cytometry data for matings between the MC4100 variants shown and the SOS reporter strain. Donors: MC4100 (plasmid-free, F-), MC4100(pED208) (WT), MC4100(pED208) mated with the SOS-uninducible *lexA3* mutant, pED208-carrying MC4100 with the gene deletions shown. Data points indicate flow cytometry events (cells) colored red to the right (SOS induced) and blue to the left (SOS uninduced) of the "red" gate, which was set using the SOS-uninducible *lexA3* mutant strain. **C.** Quantitation of the relative SOS responses exhibited by strains depicted in panel B. Results are presented as the relative SOS response, which corresponds to the ratio between the numbers of recipient reporter cells exhibiting SOS induction in matings with the MC4100*(pED208) mutant donors compared with MC4100(pED208) donors (set to 1). Values are means plus standard errors of means (SEM) (error bars). The *P* value for matings with MC4100(pED208) compared with plasmid-free MC4100 'donors' is shown (***, $P \leq 0.0005$). *P* values for comparisons between the MC4100(pED208) donor and each of the pED208-carrying MC4100 mutants were not statistically significant ($P \geq 0.05$).
